# Decoding Multi-Class Motor Imagery and Motor Execution Tasks Using Riemannian Geometry Algorithms on Large EEG Datasets

**DOI:** 10.3390/s23115051

**Published:** 2023-05-25

**Authors:** Zaid Shuqfa, Abdelkader Nasreddine Belkacem, Abderrahmane Lakas

**Affiliations:** Connected Autonomous Intelligent Systems Laboratory, Department of Computer and Network Engineering, College of IT (CIT), United Arab Emirates University (UAEU), Al Ain 15551, United Arab Emirates; 199901472@uaeu.ac.ae (Z.S.); belkacem@uaeu.ac.ae (A.N.B.)

**Keywords:** brain–computer interface (BCI), electroencephalography/electroencephalogram (EEG), motor execution (ME), motor imagery (MI), multiclass classification, Riemannian geometry decoding algorithm (RGDA)

## Abstract

The use of Riemannian geometry decoding algorithms in classifying electroencephalography-based motor-imagery brain–computer interfaces (BCIs) trials is relatively new and promises to outperform the current state-of-the-art methods by overcoming the noise and nonstationarity of electroencephalography signals. However, the related literature shows high classification accuracy on only relatively small BCI datasets. The aim of this paper is to provide a study of the performance of a novel implementation of the Riemannian geometry decoding algorithm using large BCI datasets. In this study, we apply several Riemannian geometry decoding algorithms on a large offline dataset using four adaptation strategies: baseline, rebias, supervised, and unsupervised. Each of these adaptation strategies is applied in motor execution and motor imagery for both scenarios 64 electrodes and 29 electrodes. The dataset is composed of four-class bilateral and unilateral motor imagery and motor execution of 109 subjects. We run several classification experiments and the results show that the best classification accuracy is obtained for the scenario where the baseline minimum distance to Riemannian mean has been used. The mean accuracy values up to 81.5% for motor execution, and up to 76.4% for motor imagery. The accurate classification of EEG trials helps to realize successful BCI applications that allow effective control of devices.

## 1. Introduction

A brain–computer interface (BCI) allows the brain waves of the human subject to be used to control one or more devices [1,2,3], and its importance comes from the fact that it is a control channel that requires no peripherals, nerves, or muscles to establish an interaction between the brain and machine [3,4,5,6,7]. Although there are various BCI technologies, electroencephalography (EEG) is the one used most widely by BCI researchers for reasons such as its high temporal resolution, portability, noninvasiveness, and affordability [8,9].

The past 40 years have seen advancements in the field that have led to sophisticated BCI-controlled applications [10], and many related publications and improvements have emerged recently [1,2,4,7]. Nowadays, EEG research is present in clinical settings, neuroscience, cognitive science, psychophysiology, and brain–computer interfacing, and it has potential in neuroergonomics, neurogaming, mobile brain/body imaging, entertainment, and the military [5,8,11,12,13].

However, despite its promise, real-life applications of EEG remain limited because of its low reliability in perceiving and decoding brain signals. Some of the main obstacles to accurate decoding of EEG signals are low signal-to-noise ratio (SNR), lack of calibration data [14,15,16], inter-subject and intra-subject variability of signals [4,17,18,19], and the nonstationarity of signals [12,20,21]. Efforts have been ongoing to overcome the shortcomings of EEG signal decoding, and the literature contains various preprocessing, feature extraction, and decoding algorithms designed to increase the classification accuracy of EEG recordings [21].

To design a successful BCI, two requirements are necessary; discriminative features and classification method [22]. Riemannian geometry (RG) is a robust approach for dealing with EEG readings in creating distinguishable features of the different brain states and classifying those features. It is a straightforward approach for motor imagery (MI) BCI applications. RG-based decoding algorithms (RGDAs) have attracted BCI research attention for many reasons. RGDAs are simple algorithms. That is classification is calculated based on the closeness/distance to the Riemannian mean as in the classifier Minimum Distance to Riemannian Mean (MDM) for example [23]. Despite their simplicity, RGDAs provide high accuracy as well. The main reason is that the natural geometry for MI-EEG epochs is Riemannian, not Euclidean [5,13,19,23]. In Section 2, we present a literature review of the latest advances in this area.

In MI BCI, the brain activity of the subject is sensed while they imagine moving a certain body part [4]. In this mode, kinesthetic imagination of the body part creates oscillatory activity at the sensorimotor cortex [24] without performing actual movement, i.e., motor execution (ME). These oscillations occur in certain frequency bands known as event-related synchronization (ERS) and event-related desynchronization (ERD) [3,6,25,26,27]. In terms of the regions where the ERS and ERD happen, and according to [4,28], the Sensorimotor Rhythms (SMR) for foot movement (or imagination of movement) originated in Cz and the ones for fists movements (or imagined movement) in C3 and C4. Thus, the electrodes will be affected by the SMRs differently when executing different tasks, then the signals will be ascertained. MI is also known to be spontaneous, i.e., the signals are generated by the subject intentionally without any external stimuli [4]. Unlike evoked potential signals, MI facilitates the asynchronous BCI mode of operation [9,28], and asynchronous BCI applications are user-friendly because they allow the user to generate EEG control signals voluntarily at any time without having to wait for a cue from the system.

When performing bilateral (BL) tasks in MI BCI, the subject moves (or imagines moving) both left and right limbs simultaneously i.e., both fists or feet. In contrast, the subject is asked to move (or to imagine moving) one limb either left or right at a time when performing a unilateral (UL) task. Supplementary Table S2 illustrates the tasks that are UL or BL in this study.

The recent MI BCI literature [5,12,13,14,18,19,23,24,26,29,30,31,32,33,34,35,36] reveals that the use of RGDA in MI-BCI have exploited datasets where a limited number of subjects is used. The largest dataset found in [14] contains 18 subjects where data are collected in-house and it was a mixture of motor imagery and mental imagery tasks. For the publicly available datasets, the maximum number was nine subjects from BCI Competition dataset II a. Moreover, most of the papers in the current literature do not consider the classification of the bilateral upper limbs (both fists) [5,13,23,24,26,29,32,37,38,39]. We have found that a very limited number of papers in the surveyed literature have used ME in the classification. The classification of ME reveals the real performance of the classifier because it neutralizes the subject’s level of BCI literacy, i.e., all subjects are generating SMRs in real kinesthetic activity. Please see Section 2 and Supplementary Table S1 for a comparative analysis of the current research in the field. This paper addresses the gap in the literature by exploring the performance of the RGDA static and adaptive classification for MI and ME trials on a large dataset with four BL and UL classes.

Herein, we examine the performances of different adaptation strategies of RGDAs. We exploit a large dataset (103 qualified subjects) in classifying four-class BL and UL MI and ME tasks under the following scenarios.

- We compare the performances of different RGDA implementation adaptations on BL and UL MI tasks versus BL and UL ME tasks using single-trial classification for a large dataset.
- We compare the performances of the RGDA implementation adaptations using EEG signal readings from 64 electrodes covering the scalp versus the readings from 29 electrodes covering the sensorimotor area on BL and UL MI tasks versus BL and UL ME tasks using single-trial classification for a large dataset.

The rest of this article is organized as follows. We present related work in Section 2 where a comparative analytical review of the current literature is presented. In Section 3, we describe the materials and methods used in this research including data acquisition and preprocessing, feature extraction, RGDAs, and Model training and testing. Section 4 provides a detailed description of the experiments conducted. A comprehensive discussion of the results appears in Section 5. Finally, in Section 6, we conclude and discuss future directions. Supplementary materials are also available with this paper for an in-depth analysis of our work.

## 2. Related Work

Since the inception of RGDAs in the seminal paper by Barachant et al. [23], many articles have used RG principles to classify EEG signals in MI BCIs. The typical pipeline for classifying asynchronous BCIs includes preprocessing, feature extraction, and classification (calibration and evaluation) [19], and the recent literature reveals applications of RG principles in both feature extraction and classification. Supplementary Table S1 summarizes some studies of interest that have used RG in MI BCIs.

### 2.1. Preprocessing

Preprocessing the electroencephalogram removes the randomness from the signals while retaining meaningful data [4]. It also removes unwanted signals contaminating the desired signal. The unwanted signal is called noise, interference, or artifacts. Good preprocessing increases the discriminative features in the signal, hence improving the classification performance. The signals related to relaxation, active thinking, and movement are in bands α (μ in sensorimotor context) and β. These bands are within the frequency range 8–30 Hz, where ERD and ERS exist [5,12,14,26,27]. The classical preprocessing chain for asynchronous BCIs includes frequency filtering and spatial filtering [23], but the RG approach requires no spatial filtering [14,26]. Band-pass filtering (BPF) and notch filtering (NF) are used to remove unnecessary frequencies from the readings, while epoching removes unnecessary readings by trimming the time series of the EEG trial. Epoching is also used to retain the time window of interest, and it is usually calculated from the onset of a visual (or audible) cue instructing the subject to perform a certain MI (or ME) task. In a quest for the time span in which ERS and ERD exist, many researchers have epoched EEG trials while preprocessing the EEG signals (see Supplementary Table S1).

### 2.2. Feature Extraction

Feature extraction brings out the most discriminative information within the signals, and the BCI literature suggests many feature-extraction techniques, such as time-domain, frequency-domain, time–frequency-domain, and spatial-domain ones [4]. Conventional feature-extraction methods include common spatial pattern (CSP), wavelet transform, adaptive regression model, and connectivity features such as directed transfer function (DTF), spectral coherence or phase locking values [5,24,33,40,41]. For example, CSP is an effective method for feature extraction in binary classification but performs poorly in multi-class situations; moreover, it is noise sensitive, unsuitable for small training sets [28], requires many electrodes [13], and is prone to overfitting [42].

Singh et al. [24] suggested transforming the covariance matrices (CMs) using a regularized spatial filter to increase the performance of RG classifiers while calibrating with a small sample size. The proposed spatial filter is regularized by data from other subjects and is used to reduce the dimensionality of the CMs. Known as regularized minimum distance to the Riemannian mean (R-MDRM), their method scored 20.93%, 10.65%, and 23.96% above CSP, regularized CSP (RCSP), and MDM, respectively.

Pandey et al. [33] compared the effect of RG-based feature extraction versus that of CSP on the classification performance of MI trials; they found that the former outperformed the latter when classifying using a support vector machine (SVM) and was 200% faster in training and 300% faster in evaluation. Chu et al. [31] suggested RG principles for extracting features from EEG signals: they used the Riemannian distance to extract the tangent space from the spatial CM of the EEG trial, then they used partial least-squares regression to reduce the dimensionality of the features; their experiment was successful in classifying multi-class MI tasks from the same upper limb.

Yang et al. [32] introduced the multiple Riemannian covariance multilayer perceptron (MRC-MLP) framework to classify MI tasks, which achieved 76% mean accuracy. MRC-MLP uses RG in feature extraction, where it extracts multiple CMs based on different frequency sub-bands. A multi-layer perceptron (MLP) then classifies the MI trials, but using five hidden, fully connected layers makes the model computationally expensive.

Larzabal et al. [29] introduced the Riemannian spatial pattern (RSP), using backward channel selection to make the spatial feature extraction simple and straightforward. Their approach creates a direct link between the spatial location and the Riemannian distance, which can be used to map the somatotopic arrangement of MI tasks.

In MI, the single-trial-based decoding algorithms may not gain as high accuracy as the repetition of the trials [43]. However, it is more efficient and yields faster decoding.

### 2.3. Classification

An accurate decoding algorithm is vital for creating a reliable BCI application [12,19,44]. Recently, classification methods that deal with the intrinsic geometry of the spatial CMs (SCMs) have received more attention in BCI research [12,13], and RGDAs have been proposed to neutralize the flaws in EEG signals and increase the decoding performance [4,12,19,35]. Yger et al. [12] and Kumar et al. [14] suggested that because RGDAs are known to be adaptive, they can overcome the effects of the noise and nonstationarity of EEG signals. The adaptive RGDAs can deal with signal variability as they adjust over time [45]. There are three common adaptation methods; supervised, and unsupervised, and rebiased [40,46]. In their review, Congedo et al. [19] regarded the use of RG in BCIs as simple, accurate, and robust. It is mathematically sharp and algorithmically simple, which may make it suitable for online decoding algorithms, and furthermore, it has a high transfer-learning capability.

Guan et al. [13] proposed a new classification framework and data-reduction methods for classifying MI tasks on three different datasets with three and four classes (tasks). The datasets comprised nine, three, and seven subjects. They used a subject-specific decision tree (SSDT) framework with filter geodesic minimum distance to Riemannian mean (FGMDM) to classify MI trials. Kumar et al. [14] classified MI trials (four classes) and nonmotor mental-imagery trials (three classes) using RG. They introduced five different adaptation strategies for each of the Minimum Distance to the Riemannian Mean (MDM) and the Fisher geodesic Minimum Distance to the Riemannian Mean (FgMDM). However, they investigated the performance on only two datasets, the first of which was a small dataset, and the second comprised only three classes (only one of which was MI). See Supplementary Table S1 for the performances of the different classifiers and Section 3.3 for more about the adaptation strategies. Ju et al. [47] tested the performance of MDM in a federated transfer-learning setting with a large dataset, but their experiment addressed binary MI classification and achieved only 60% accuracy in subject-specific results.

Despite the effective novel application of RGDAs brought to the BCI field, the review papers [4,12,19,40] have listed a couple of opportunities where the Riemannian-based solutions can be improved. The performance of the classifier may decrease when using a large number of electrodes where the class prototype CMs call for more samples to build. The lack of a stable estimator of the Riemannian median is still a challenge for the people in the domain which may lead to a more robust RGDA classification. RGDAs have not been tested in scenarios of transfer learning. Furthermore, RGDA is still tested in an offline, in-the-lab controlled environment, and it is not clear yet how they will perform in real-life online settings. In terms of complexity, RGDAs are computationally expensive with an order of growth of $O(n^{3})$ where *n* is the count of electrodes. Finally, the increase in dimensionality makes the calculation of the distance more sensitive to noise.

Gao et. al [37] have suggested using the Riemannian Geometry characteristics to map the extracted time-domain and spatial features on a Riemannian manifold to suppress extreme values and neutralize noise. The features were extracted using CNNs, mapped into an SPD and finally classified using fully connected NN. The reported average accuracy is 82.10%. However, their dataset is limited (nine subjects) with four MI classes (left hand, right hand, both feet, and tongue) and there were no ME tasks.

Another paper has fused deep learning and RGDAs as well. The average accuracy of the proposed model in [38] is 80.63%. They have used the BCI Competition IV-2 dataset just like the paper above [37]. This dataset includes only nine subjects.

In [39], a comparison between the performance of MDM using conventional covariance matrix extraction and the use of sliding window with different sliding rates and window widths has been presented. They also used the filterbank approach and it shows an increase in the classification accuracy. The reported average accuracy (76.09%) is still below the performance in this paper and the dataset is limited to nine with four classes as well.

Many papers in the literature have worked on the same dataset. However, they have not demonstrated the performance of the RGDAs on such a large dataset. For example, in [48], Abenna et al. have achieved MI classification accuracy above 95% for multiclass and binary classification. They have used a combination of methods to increase the accuracy. They have reduced the noise by using Delta rhythms. CSP has been used for feature extraction. A decision tree is used for feature selection. Light Gradient Boosting Machine (LGBM) has been used for classification where Particle Swarm Optimization (PSO) is used to fine-tune the classification parameters. Different combinations of classes were tested; combinations of four classes and two classes every time. In addition, they have demonstrated that the selection of a small number of channels, as low as four channels in binary classification may not degrade the performance a lot. Although they have experimented with their proposed model on the same dataset, they have chosen to run their classification on MI only. Moreover, the proposed methods show significant improvement in classification accuracy, they have not been evaluated on Riemannian classifiers. They have stated that they have chosen LGBM because of its superior performance compared to other classifiers without mentioning those classifiers. Moreover, the proposed work has experimented with a smaller number of electrodes (58, 16, 14, 9, 3) for binary classification, not multiclass classification. In another experiment, they demonstrated the performance of multi-class classification with 44 electrodes. However, two of the classes were baseline classes, not MI. Unlike our proposed work, where we experimented with Imagine Left Fist, Imagine Right Fist, Imagine Both Fists, and Imagine Both Feet, their classes were Imagine Both Fists, Imagine Both Feet, eyes open (baseline), and eyes closed (baseline).

In their paper, Nisar et. al. [49] have extracted five features from the EEG signal using sliding window methods. The features are Band Power (BP), Approximate Entropy (ApEn), statistical features, wavelet-based features, and Common Spatial Pattern (CSP). Then, Decision Tree (DT), Random Forest (RF), Support Vector Machine (SVM), K-Nearest Neighbors (KNN), and Artificial Neural Network (ANN) are used to classify the trials. Their best classification accuracy was 98.53% SVM binary classifier. Though they have not demonstrated the performance of the RGDAs on the dataset. In addition, they used part of the dataset only (50 subjects) and for binary classification (Left hand and right hand). Moreover, they have not experimented on channels less than 64.

Interesting work has demonstrated the effectiveness of the Deep Autoencoder (DAE) and CNN to decode the EEG signal on the same dataset in [50]. They have used a variational autoencoder (VAE) to denoise the signals before being decoded. Although the method shows a decent classification accuracy (97.72%) for four-class MI decoding, it has used 10 subjects only and has not used Riemannian geometry characteristics.

In [51], the authors have demonstrated the performance of three classifiers: Linear Discriminant (LD), Naive Bayes (NB), and SVM for classification. They have used 30 statistical features to represent the EEG signals, and they classified the statistical feature vector. The SVM has achieved the highest performance with 99.51% accurate classification (averaged for 96 subjects). One downside of this study is that it is not clear whether the six classes were specific MI or ME trials or the signal recording data structures themselves. If they are the MI tasks, they should be any of Imagine Left Fist, Imagine Right Fist, Imagine Both Fists, Imagine Both Feet, Execute Left Fist, Execute Right Fist, Execute Both Fists, and Execute Both Feet. The paper instead presents six classes. Those classes are eyes open, eyes closed, open and close left or right fist, imagine opening and closing left or right fist, and open and close both fists. Not to mention that the paper has not come across Riemannian Geometry at all as well. Their chosen approach, the way they specify the classes, may not be practical for BCI applications, from our point of understanding.

A comparison among different classifiers, for binary classification (Movement vs. Relax), has been presented in [52]. They have compared the performance of, Support Vector machines (SVM), K-nearest neighbor (KNN), Quadratic Discriminant Analysis (QDA), Linear Discriminant Analysis (LDA), Naive Bayes (NB), and Ensemble in binary classification. They have chosen to classify execution and imagery of upper limb vs inactivity trials, not the MI or ME trials among themselves, i.e., any of Imagine Left Fist, Imagine Right Fist, Imagine Both Fists, and Imagine Both Feet or Execute Left Fist, Execute Right Fist, Execute Both Fists, and Execute Both Feet. They have extracted six powerbands and four time-frequency domains as features and have achieved 100% accuracy for NB and QDA. They have exploited the EEG signal of 10 subjects with only upper limp MI and ME as well, not the full dataset with its four classes for each of type of task (ME or MI).

## 3. Materials and Methods

### 3.1. Dataset, Experimental Paradigm, and EEG Signal Preprocessing

The dataset used in this study was the EEG Motor Movement/Imagery Dataset v1.0.0. The original dataset comprises more than 1500 EEG recordings taken from 109 volunteered subjects. Each subject has 26-min EEG recordings in European Data Format ’plus’ files (EDF+) [53,54].

To prepare the data for classification, they were pushed through a pipeline of data munging, cleansing, preprocessing, and feature extraction. The data were accessed and downloaded using WFDB Toolbox for MATLAB and Octave [55,56,57]. The data were read and stored in a MATLAB-accessible format as pairs of signal and annotation matrices. The signal matrix comprised the electrode readings over time, making it of size (datasamples×(electrodes+1)); the corresponding annotation matrix indicated the annotation and the duration of each trial, and its size was (trials×2).

Some files were found not to comply with the description of the data-collection experiment in Supplementary Materials (Dataset Description and Experimental Paradigm), i.e., files missing some trials or run files that were shorter than expected. Any subject with files with issues was discarded from our experiment; e.g., subjects S088, S089, S092, S100, S104, who had run lengths that were less than expected. The subjects included in the present study were S001–S087, S090–S091, S093–S099, S101–S103, S105, and S107–S109. The subjects were then renumbered from 1 to 103 while maintaining the original sequence.

From the annotation matrix and using the trial duration, the onset and end of each trial were calculated, and the unannotated readings after the end of the last trial were discarded from each run. Finally, the 65th column was dropped while retaining the signals from the 64 electrodes. The annotations T0, T1, and T2 in the annotation matrix were also used to label the trials.

The signals were filtered using a fifth-order Butterworth band-pass filter (BwBPF) (8–30 Hz) to retain SMR discarding other frequencies that are undesired, then the trials from each run were epoched from the onset to 4.0 s, resulting in 640 signal readings per channel per trial. As we are decoding offline tasks, we have used common epoch length (see Supplementary Table S1) to obtain the best performance of our classification. They were then ordered per subject per trial nature (ME/MI) in two three-dimensional matrices of size signals×electrodes×trials(640×64×90), one for ME trials and one for MI trials, discarding the relax trials. Later, the trials were grouped by type and class: ME left fist, ME right fist, ME both fists, ME both feet, MI left fist, MI right fist, MI both fists, and MI both feet.

### 3.2. Feature Extraction

EEG trials are time windows of EEG readings, and summarizing the former in SCMs has proved successful in BCI applications [23,26]. The SCMs are symmetric positive definites (SPDs) that exist in a Riemannian manifold. For a given EEG trial *T*, there is a set *C* of channels (electrode readings) that record the data on intervals based on sampling rate. The length of the time window in seconds multiplied by the frequency gives the number *S* of data samples in the trial, and the trial can be represented as a matrix *E* of size S×C. It has been suggested [5,14] that the CM of each EEG trial is considered as the SPD of that epoch, which can be estimated as

(1) $$CM=\frac{EE^{T}}{tr(EE^{T})}.$$

**Figure 1:**
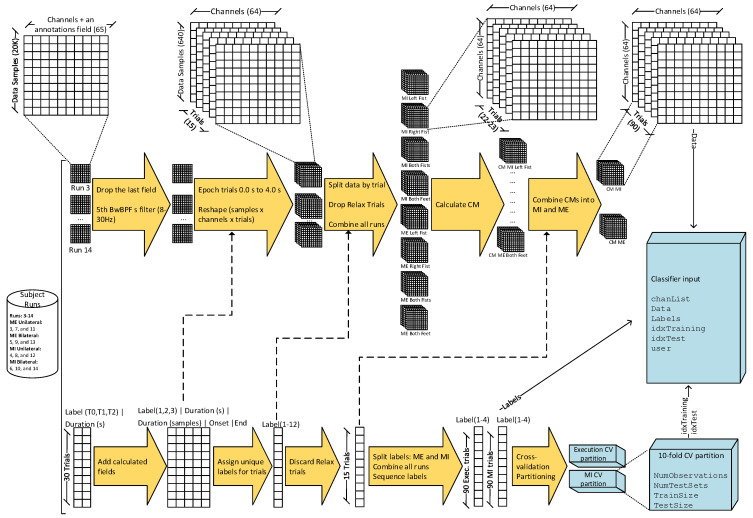
Preprocessing and feature-extraction pipeline: The data are cleansed, preprocessed, classified, and labeled. Features are extracted using CM estimator before training the classifiers. The data are also partitioned using 10–fold cross–validation.

We used the function covariances proposed by [23] to estimate the CMs of each set of trials, resulting in matrices of size electrodes×electrodes×trials, where each SCM represents a trial. Finally, the CMs were gathered per subject into two three-dimensional matrices of size electrodes×electrodes×trials(64×64×90), one for ME trials and the other for MI trials. Figure 1 shows the data wrangling and preprocessing used to prepare the data for classification.

To compare the results of the classification of EEG readings from the whole scalp (64 electrodes) versus the readings from the electrodes over the sensorimotor area, we dropped the rest of the channels. Channel readings from the electrodes over the sensorimotor cortex [28] (FC,C,CP,FT,T,TP) were extracted, and the resulting data were pushed through the same pipeline. The resulting dataset is referred to as the sensorimotor area electrodes or readings from 29 electrodes onward. Readings from ME trials and MI trials were segregated and referred to as ME tasks and MI tasks, respectively. Thus, we ended up with the following datasets: MI tasks from 64 electrodes, MI tasks from 29 electrodes, ME tasks from 64 electrodes, and ME tasks from 29 electrodes.

### 3.3. RG-Based Decoding Algorithms

The current literature suggests that Euclidean space is unsuitable for dealing with the SPDs of the different epochs of EEG signals, and the natural geometry for EEG signals is Riemannian space [5,13,19,23]. According to Barachant et al. [23], the Riemannian distance between the SPDs of reference signal epochs and other signal epochs can be used to classify the signal epoch. However, Barachant et al. [26] stated that latent Dirichlet allocation, SVMs, neural networks, and other efficient classification algorithms could not be implemented directly in a Riemannian manifold thus methods that depend on Euclidean space have issues regarding stability and accuracy [5]. Instead, the Riemannian mean uses the geometric properties of SPDs to discriminate among different CMs [24].

The classification used herein employs different combinations of classifiers and adaptation strategies as suggested by [14,46] MDM and FgMDM, i.e., baseline MDM (MDM), supervised MDM (MDMS), unsupervised MDM (MDMU), rebias MDM (MDMR), supervised MDMR (MDMRS), baseline FgMDM (FgMDM), supervised FgMDM (FgMDMS), unsupervised FgMDM (FgMDMU), rebias FgMDM (FgMDMR), supervised FgMDMR (FgMDMRS), unsupervised MDMR (MDMRU), and unsupervised FgMDMR (FgMDMRU).

The supervised, unsupervised, and rebias adaptation strategies simulate the calibration and evaluation of the classifier as if it is operating in online MI BCI. In supervised adaptation, the classifier, the class prototype, or parameters are updated based on the ground truth of the testing trial after each classification, whereas unsupervised adaptation uses the incoming trial SPD to update the class prototype after each prediction [14]. Finally, rebias adaptation shifts the classifier output to rectify the change that appeared in the data distribution because of the inter-session and inter-subject nonstationarity variability. In some combinations, the classifier and the class prototype are updated to reflect the change [14,46].

**Figure 2:**
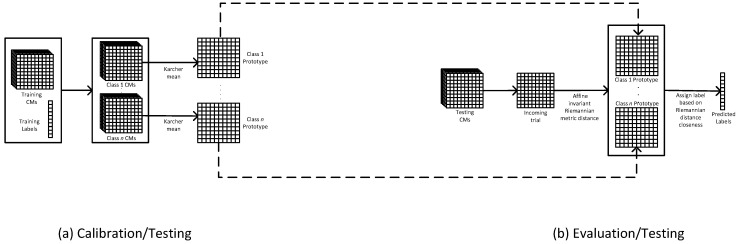
MDM classifier: (**a**) calibration of the classifier using CMs of testing data; (**b**) evaluation of the classification performance. For the schematic diagrams of the rest of the classifiers with their adaptation strategies, please refer to the Supplementary Materials.

The MDM is the simplest Riemannian approach [19]. To classify trials with the MDM, the CMs from calibration data are used to estimate a class prototype, one per class. Then, the Riemannian distance between the class prototype and the CM of each trial from the evaluation data is measured. Finally, the trial is assigned a label based on its closeness to the prototype [14,23,29]. See Figure 2 for the schematic diagram of MDM.

On the other hand, FgMDM works exactly the same except that it generates a discriminant Fisher geodesic (Fg) filter and reference CM from the SPDs before training the MDM [13,14,23,24,58]. Then, the reference CM and the Fg filter are used to project the training trials onto the tangent space and filter them. See Supplementary Figure S10: FgMDM Schematic Diagram. The Fg filtering helps when there is a large distance between the calibration SPD (class prototype) and the testing SPD, which may be caused by noise, i.e., not native to the class per se [23].

In the MDMS adaptation strategy, the calibration goes exactly as in MDM, except that the class prototype is updated after each classification of a trail. The class prototype corresponding to the actual label of the trial is updated with the trial CM. FgMDMS is built on the FgMDM adaptation strategy. It borrows the same update mechanism as in MDMS, except that instead of updating the class prototype immediately, it updates the reference CM and the Fg Filter using the current predicated trial and its actual label. Likewise, MDMU and FgMDMU adapt except that the update is based on the predicted label, not the ground truth of the incoming trial.

MDMR (Supplementary Figure S7) uses the MDM classifier but adapts by shifting the data using the reference matrix, i.e., not updating the class prototype. Reference CM for the calibration trials is calculated using Karcher mean. The resulting reference matrix is used to shift the calibration CMs before calculating the class prototype. It is updated each time an incoming testing trial is going for prediction, which in turn is shifted using the updated reference matrix before each prediction. The same applies to FgMDMR (Supplementary Figure S13), where the FgMDM classifier uses the same rebias mechanism. The reference matrix calculated from training data is used to transform them back. Before each prediction, the reference matrix is updated with the incoming trial and then transforms the incoming trial before it goes into filtering and classification.

For the classifiers MDMRS and MDMRU, the adaptation happens on two levels, the reference CM is updated before each prediction, and the class prototype is updated after each prediction; based on the actual label in MDMRS or on the predicted label in MDMRU.

FgMDMRS and FgMDMRU in turn are similar to MDMRS and MDMRU except for the fact that they are built on FgMDM classifier, not MDM.

See [14] for more details about the mathematical modeling and the adaptation algorithms of the different strategies. For the schematic illustrations of the classifiers, please refer to Supplementary Figures S4–S15 in the Supplementary Materials.

### 3.4. Model Training and Testing

Because of the small number of examples (90 trials), ten–fold stratified cross–validation (CV) was used to calibrate and evaluate the performance of the model. The number of examples per class is identical (22–23 examples per class). The model training and testing took place in MATLAB (R2021a) Update 3 on Windows 10 64-bit Home edition. The processor was AMD Ryzen 7 4800H with Radeon Graphics 2.90 GHz and 16.0 GB of RAM.

## 4. Results

In this section, we present the results obtained from executing the different RGDA adaptation strategies on the dataset explained above. We ran 12 different adaptations of the RGDAs (see Section 3 for details) in four different settings. The classifiers were used to classify the trials from 29 and 64 electrodes for both ME and MI. The performances of the classifiers are summarized in Table 1, where the mean classifier accuracy and standard deviation among the 103 subjects are given.

Three-way repeated-measures analysis of variance (three-way RM ANOVA) was conducted to determine the effects of electrode placement, nature of the task, and classifier adaptation on the accuracy, i.e., Electrodes*Task*Classifier (Electrodes: 29 or 64; Task: ME or MI; Classifier: MDM, MDMS, MDMU, MDMR, MDMRS, MDMRU, FgMDM, FgMDMS, FgMDMU, FgMDMR, FgMDMRS, or FgMDMRU; dependent variable: accuracy).

Although there was no statistically significant three-way interaction among Electrodes*Task*Classifier, i.e., F(4.225,430.960)=1.344,p=0.251,ϵ=0.384 (using Greenhouse–Geisser correction for sphericity), there was a statistically significant two-way Electrodes*Classifier interaction, i.e., F(3.548,361.873)=20.284,p<0.0005,ϵ=0.323, and two-way Task*Classifier interaction, i.e., F(2.763,281.840)=45.811,p<0.0005,ϵ=0.251. The two-way Electrodes*Task interaction was not statistically significant, i.e., F(1.000,102.000)=0.0620p=0.804.

Then, the simple main effect with Bonferroni correction was used to compare the performances of the classifiers in different combinations, as elaborated in the subsections below. In the following subsections, we present the pairwise comparison of the performances of the classifiers from two perspectives. First, we compare the performances of the same adaptation under different settings in Section 4.1, Section 4.2, Section 4.3 and Section 4.4. Second, in Section 4.5, we compare one adaptation performance in front of all other adaptations while maintaining the same settings, i.e., the number of electrodes and nature of the task. Summarizing of the results can be sought in Table 1 and Supplementary Figure S2. For deeper statistical insights, see the Supplementary Materials. For ease of traceability and to highlight patterns, we use a unified convention in the tables summarizing the results of the statistical tests. In Supplementary Tables S4–S11, significant values are emphasized in bold italics, negative values are in red, all values are with the 95% confidence interval, and the significance level is p<0.01; all the statistics were calculated using SPSS.

Also, classification performance per class and confusion matrices for all classifiers appear in the Supplementary Materials. Please see Supplementary Figures S16–S27 for per-class performance and Supplementary Figures S28–S39 for confusion matrices.

### 4.1. Sensorimotor Area (29 Electrodes) vs. 64 Electrodes with Motor-Execution Tasks

In Supplementary Table S10, we summarize the pairwise comparison of all classification adaptation performances for the ME trials when using 29 electrodes versus 64 electrodes; Supplementary Figure S2 depicts the pairwise comparison for quick reference. From the table, except for FgMDM, FgMDMR, FgMDMRS, and FgMDMRU, it is clear that the performances of all adaptations when classifying ME trials from 29 electrodes are significantly poorer than those when classifying the same trials from 64 electrodes. For FgMDM and FgMDMR, FgMDMRS, and FgMDMRU, their performances are also poorer but not significantly so. FgMDMS scored slightly higher with 29 electrodes than with 64 electrodes, but the difference is not statistically significant.

### 4.2. Sensorimotor Area (29 Electrodes) vs. 64 Electrodes with Motor-Imagery Tasks

Supplementary Figure S2 and the Supplementary Table S11 present the results of the post-hoc test between the classification performances of MI trials from 29 electrodes and 64 electrodes. It is evident that the mean accuracy of all classifiers is significantly lower when using 29 electrodes compared to using 64 electrodes. The difference is between 4.94 and 1.46 for MDMU and FgMDMS, respectively.

### 4.3. ME vs. MI Tasks (29 Electrodes for Sensorimotor Area)

When comparing the performances of the classifiers while classifying trials from 29 electrodes, and from the Supplementary Table S8 and Supplementary Figure S2, an interesting pattern emerges. The baseline MDM and its supervised and unsupervised adaptations (MDMS and MDMU) performed statistically significantly better when classifying ME trials compared to when classifying MI trials. FgMDM and its supervised and unsupervised adaptations (FgMDMS and FgMDMU) performed slightly better for ME compared to MI, but the difference was not statistically significant. In contrast, all the rebias adaptations (MDMR, MDMRS, MDMRU, FgMDMR, FgMDMRS, and FgMDMRU) performed better when classifying MI compared to ME, and the difference was statistically significant except for FgMDMR and FgMDMRU.

### 4.4. ME vs. MI Tasks (64 Electrodes)

The pattern discussed in the previous subsection becomes clearer when comparing the classifier performances with 64 electrodes. MDM, MDMS, and MDMU performed better when classifying ME compared to MI, with a difference that was statistically significant. For all other adaptations, the performance for MI was higher than that for ME; see Supplementary Figure S2 and Supplementary Table S9 for the significance information.

From the pairwise comparison analyses in Section 4.3 and Section 4.4, it is evident that MDM, MDMS, and MDMU perform significantly better when classifying ME trials compared to MI trials from both 64- and 29-electrode readings. Furthermore, Supplementary Tables S8–S11 show that 36 of the 48 pairwise comparisons exhibited a significant difference. We expected the performances to be almost the same, given that the literature suggests that imagining movement creates sensorimotor rhythms (ERD and ERS) just like if the subject had executed the movement virtually [6]. In addition, the literature suggests that BCI-literate subjects can produce high-quality sensorimotor rhythm oscillations [27].

### 4.5. Comparison of Classifiers

Simple main effect and pairwise comparison were used to compare the performances of the classifiers in the four settings of ME 29 electrodes, MI 29 electrodes, ME 64 electrodes, and MI 64 electrodes. When classifying the readings of ME trials from 29 electrodes, we used Greenhouse–Geisser correction, which showed statistically significant differences among different classifier adaptations, i.e., F(2.384,243.145)=2413.529,p<0.0005,ϵ=0.217. The post-hoc tests using Bonferroni correction were as given in Supplementary Table S4.

Supplementary Table S5 presents the pairwise comparison of the classification mean accuracies while classifying the MI trials from 29 electrodes. The Greenhouse–Geisser correction showed statistically significant differences among different classifier adaptations, i.e., F(2.420,246.876)=2284.137,p<0.0005,ϵ=0.220.

Supplementary Table S6 presents the pairwise comparison of the mean accuracies of the different RGDAs in the ME trials from 64 electrodes. The Greenhouse–Geisser correction showed statistically significant differences among the different classifier adaptations, i.e., F(2.131,217.386)=2209.239,p<0.0005,ϵ=0.194. The post-hoc tests using Bonferroni correction were as given in Supplementary Table S6.

Supplementary Table S7 presents the results of the post-hoc test for the MI 64-electrode setting. Using Greenhouse–Geisser correction, there were significant differences among the different classifier adaptations, i.e., F(2.076,211.746)=1802.822,p<0.0005,ϵ=0.189.

**Figure 3:**
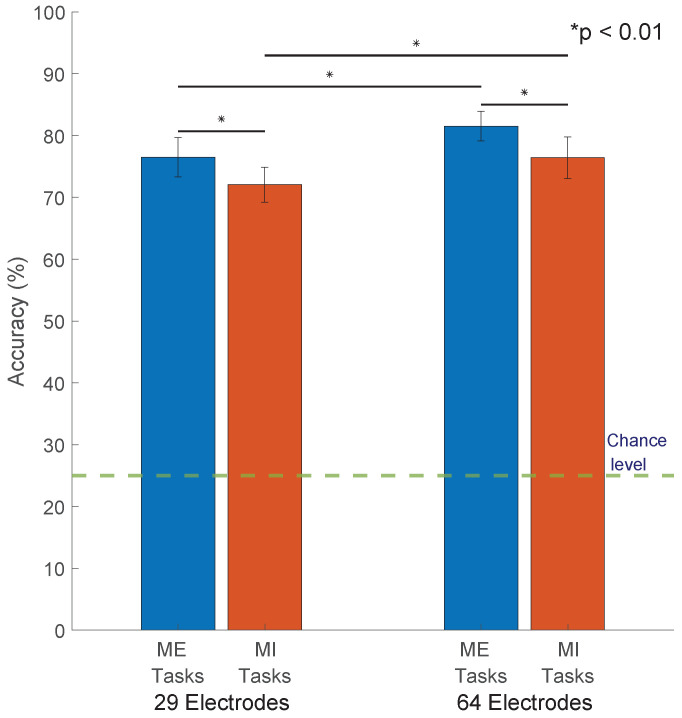
MDM classifier accuracy: The Classifier Minimum Distance to the Riemannian Mean (MDM) performed better than the other classifiers under all scenarios. The performance of the MDM classifiers are ME 29 electrodes (76.483 ± 3.174 with lower and upper bounds 75.863 and 77.104), MI 29 electrodes (72.050 ± 2.825 with lower and upper bounds 71.497 and 72.602), ME 64 electrodes (81.521 ± 2.400 with lower and upper bounds 81.052 and 81.990), and MI 64 electrodes (76.419 ± 3.355 with lower and upper bounds 75.763 and 77.074). All upper and lower bounds are at 95% Confidence Interval. The error bars represent ± one standard deviation. The differences between the performance of the classifiers are statistically significant (see the significance bars spanning over the bar chart). Since it is a 4-class classification, the chance level is 25%.

From Supplementary Tables S4–S7, we notice some patterns and noteworthy trends. The baseline RG classifier MDM and its supervised and unsupervised adaptations MDMS and MDMU outperformed the other classifiers when compared in the same scenarios, i.e., number of electrodes (29 or 64) and trial nature (ME or MI). MDM had the highest accuracies of 76.48%, 72.05%, 81.52%, and 76.42% when classifying 29-electrode ME trials, 29-electrode MI trials, 64-electrode ME trials, and 64-electrode MI trials, respectively. Figure 3 shows the mean accuracies of MDM in all scenarios, and Supplementary Figure S3 shows how it performs among different subjects. MDMS scored 80.70%, 75.39%, 75.07%, and 70.50% for 64-electrode ME, 29-electrode ME, 64-electrode MI, and 29-electrode MI, respectively, and the accuracy of MDMU was 80.33%, 74.77%, 73.97%, and 69.83% for 64-electrode ME, 29-electrode ME, 64-electrode MI, and 29-electrode MI, respectively. The obtained accuracies are at the minimum requirement for reliable BCI control, which is 70% as stated in [43].

The supervised adaptation strategies outperformed the unsupervised ones. However, the difference was statistically significant when classifying trials using only 29-electrode readings for both tasks (MI and ME).

Unlike the results in [14], applying the Fg filter to the SPDs did not enhance the performance of the classification in the baseline, supervised, and unsupervised adaptation strategies. Supplementary Tables S4–S7 and Supplementary Figure S2 shows that FgMDM, FgMDMS, and FgMDMU were significantly less accurate than MDM, MDMS, and MDMU. In addition, the rebias adaptation strategy lowered the performances of the MDM, MDMS, and MDMU classifiers; see Supplementary Figure S2 and supplementary Tables S4–S7.

## 5. Discussion

Although the Fg filter is ideal for dealing with trials contaminated with noise because of its ability to discriminate their SPDs in Euclidean space, the performances of the classifiers with Fg adaptation were lower than those of MDM (with no Fg filter) in all scenarios. There are two possible reasons for this drop in classification performance: (i) the classifier suffers from overfitting; (ii) the Fg filter distorts the features among the SPDs while mapping Riemannian space into Euclidean space or vice versa. By looking at the performance per class bar charts (Supplementary Figures S22–S24) and the confusion matrices (Supplementary Figures S34–S36) of the Fg adaptation, we can find noteworthy observations. Although classes 1 and 2 can be distinguished accurately from classes 3 and 4 by the classifier, the classifier confuses when deciding whether the class is 1 or 2. The same applies to classes 3 and 4. For example, In the FgMDM classifier (ME 64 electrodes), about 84% of False Positive predictions of class 1 come from class 2, and about 89% of the False Negative predictions of class 1 go to class 2. Class 2 suffers from the same observation against class 1 as well. The classification of classes 3 and 4 shows the same behavior. It is even more interesting when you can see that class 1 is predicted correctly as class 1 (True positive) 920 times, whereas it is falsely predicted as class 2 1178 times. The opposite is also present where class 2 is TP in 865 predictions and falsely predicted as class 1 in 1214 predictions. Classes 3 and 4 show the same pattern too.

It seems that the Fg adaptation when projecting into Euclidean space, filtering, and de-projecting back into Riemannian space, the trials’ CMs, distorts the features of the classes that are close to each other. Again, the pattern persists in all pure Fg adaptations (FgMDM, FgMDMS, and FgMDMU), and it is milder with Fg Rebias adaptations (FgMDMR, FgMDMRS, and FgMDMRU) but still apparent. In our experiment, the Fg classifiers mix the UL classes (left fist and right fist) and the BL classes (both fist and both feet). Further investigation is required to assess the effectiveness of the Fg filter on the performance of the classifier for this dataset versus that used in [14]. We anticipate that using the Fg classification as a series of binary classifiers to classify the multi-class datasets may overcome this problem. We suggest a binary classifier to discriminate UL and BL trials, followed by two binary classifiers (one for discriminating Class 1 from Class 2 and the other for Classes 3 and 4).

The low performance of the rebias adaptation strategy could be because retrain and rebias strategies were developed to deal with inter-session and inter-subject variabilities, whereas in our case, the trials were from the same subject and the same session. Another reason could be the use of cross–validation to calibrate and evaluate the performances. Shenoy et al. [46] concluded that if the data are fairly stable but not highly nonstationary, then the baseline cross–validation error will be lower than the rebias adaptation cross–validation error. In the same context, Kumar et al. [14] used session-to-session calibration and evaluation trials rather than cross–validation when experimenting with different adaptation strategies, including rebias. Furthermore, rebias adaptation was designed to adapt the classification of BCI signals in an online setting [46] where the trials took place in session after session or subject after subject, where cross–validation is neither practical nor possible. Finally, the baseline performs better due to the insufficiency of the data to train the methods with adaptation strategies [46].

The supervised adaptation MDM performed lower than the baseline because updating the class prototype after each classification increases the fitness of the classifier, i.e., the more the model tunes the prototype, the more the class prototype becomes specific; the same applies to the unsupervised adaptation. Another reason that may pertain to the unsupervised adaptation performance is that a misclassified trial contributes adversely when updating the class prototype.

The dataset description and experimental paradigm did not elaborate on the level of BCI literacy or training of the subjects. The subjects are described simply as 109 volunteers, and we anticipate that it would be challenging to secure 109 BCI-literate subjects. Therefore, we can relate the difference in the performance of the classification results between ME and MI to the fact that the subjects were BCI-naive. Thus, their ability to produce sensorimotor rhythms (ERD and ERS) while imagining the movement was not as consistent as when they were executing the movement virtually.

We anticipated that the selection of the electrodes might reduce the noise when only the channels of interest are present in the trials. A possible reason for this is that the placement of the EEG electrodes over the designated areas is approximate because of many factors, including anatomical and physiological differences among subjects; instrumental factors may be present as well.

The Information Transfer Rate (ITR) using the proposed algorithms is 15 bits/min which may not look suitable for real-time BCI applications [22]. However, the average classification accuracy of the proposed algorithm was higher than 70% which is the minimum requirement for developing real-time BCI applications.

Generally, RGDAs perform better when the number of electrodes is relatively small. As the number of channels increases, the algorithm needs more samples to estimate the class prototype accurately. In addition, they consume more computation resources since they are computationally complex. In addition, the increase in the number of electrodes degrades the calculation of the Riemannian distance. Despite the aforementioned pros, RGDAs are simple in concept and provide relatively high accuracy. Unlike other conventional feature extraction and classification methods, they work on the Riemannian space, the native space for EEG signals [5,19,23].

Different adaptation methods bring a variety of benefits, definitely at a cost. The adaptation strategies rebias, supervised, and unsupervised adapt to the incoming trials while testing (or using in an online setting), unlike the baseline classifier which does not update while testing. Hence, the baseline classifier is known to be static [40]. After the calibration, the adaptive classifiers can fine-tune their parameters as they are classifying incoming EEG trials. That adaptation helps dealing with the known issues of non-stationarity and variability of EEG signal [45]. The low performance of the adaptation strategies compared to the baseline can be regraded to data insufficiency where adaptive classifiers require more data examples to adapt properly [46]. Add to this, some adaptation strategies do not exist in practice regularly, i.e., the supervised ones, unless the design of the BCI captures the user feedback. For a full comparison of the different adaptation strategies, please see Supplementary Materials, Supplementary Table S3).

## 6. Conclusions

The current literature addressing the application of RGDAs in MI BCI classification lacks experiments on large datasets, does not investigate the classification of BL MI tasks, and in many cases, addresses only binary classification. Herein, we investigated the accuracy of 12 different RGDA variations on 103 subjects with four-class UL and BL MI and ME tasks where the trials were extracted from 64 electrodes and 29 electrodes. The classifiers were MDM and FgMDM with different adaptation strategies, i.e., supervised, unsupervised, and rebias ones. The baseline MDM recorded the highest accuracies of 81.52%, 76.48%, 76.42%, and 72.05% for 64-electrode ME, 29-electrode ME, 64-electrode MI, and 29-electrode MI, respectively. We suggest using the baseline classifier and its supervised and unsupervised adaptations when there are no high inter-session and inter-subject variabilities where the Fg filtering and the rebias adaptation strategies may degrade the performance of the MDM classifier. On top of that, the MDM classifier is simpler in concept and less expensive computationally. It is known in the literature that, the rebias adaptive strategy calls for more training examples when the data are highly dimensional [46]. In addition, the rebias classifiers outperform the static classifiers only when the data variability is low, i.e., the data are stable. The classification in the paper is conducted on trials from the same subject and same session which makes the data more stable and it justifies the low performance of the rebias adaptation strategy.

Our proposed method does not take into consideration the onset of the movement, epoching is used instead, which makes it suitable for offline experimentation. However, the decoding time during the testing is relatively short; on average, the MDM classifier spent 5.3755 × 10${}^{-3}$ s. classifying each trial from Subject 1. The efficiency of the classification makes it practical to be used in the online setting. The only adjustment required is adding a sliding window to detect movement onset.

The work in this paper may be extended to investigate the strategies to deal with the overfitting of the classifier and the feature distortion when using the Fg filter adaptation. In addition, comparing the algorithms in this study against the state-of-the-art algorithms using performance measures other than accuracy; comparing efficiency and information transfer rate may be an interesting direction as well.

Many future directions remain to be addressed. The size of the dataset makes it a good repository for experimenting with transfer learning and federated learning for MI BCI RGDAs, and a large number of subjects offers an opportunity for subject-to-subject transfer learning. Furthermore, EEG feature fusion could be investigated to reduce the dimensionality of the EEG signals rather than selecting certain channels. This might reduce the computational resources required and provide better classification performance by concentrating on the channels where discrimination features exist. The multi-class decoding with single-trial is still an attractive area of research. The non-stationarity of EEG signals among subjects and between sessions makes it an open challenge for more research to develop reliable yet efficient decoding methods. It would also help to unify the learned trial features in federated EEG learning when heterogeneous systems are used among the edges. Another interesting future direction would be to use the dataset to test the classification accuracy of neural networks or deep-learning classifiers.

## Figures and Tables

**Table 1:** Classification accuracy for readings from 29 electrodes vs. 64 electrodes and for motor imagery (MI) vs. motor execution (ME) tasks.

		29 Electrodes				64 Electrodes		
	**ME Tasks**		**MI Tasks**		**ME Tasks**		**MI Tasks**	
	**Mean**	**Std.**	**Mean**	**Std.**	**Mean**	**Std.**	**Mean**	**Std.**
**Classifier**	**Accuracy**	**Deviation**	**Accuracy**	**Deviation**	**Accuracy**	**Deviation**	**Accuracy**	**Deviation**
**MDM**	0.76483	0.03174	0.72050	0.02825	0.81521	0.02400	0.76419	0.03355
**MDMS**	0.75394	0.03342	0.70496	0.02693	0.80701	0.02873	0.75070	0.02971
**MDMU**	0.73970	0.03661	0.69827	0.02804	0.80334	0.03436	0.74768	0.03229
**MDMR**	0.31348	0.04985	0.33085	0.03849	0.34401	0.04615	0.35750	0.03532
**MDMRS**	0.30043	0.04841	0.31866	0.03409	0.33290	0.04340	0.34800	0.03438
**MDMRU**	0.29633	0.04330	0.31715	0.03547	0.32902	0.04321	0.34520	0.03058
**FgMDM**	0.39827	0.06295	0.39666	0.06615	0.41241	0.07459	0.41446	0.07614
**FgMDMS**	0.39331	0.06716	0.38749	0.06767	0.39482	0.07815	0.40216	0.07624
**FgMDMU**	0.39687	0.06075	0.39353	0.06523	0.41413	0.06969	0.41564	0.07025
**FgMDMR**	0.33517	0.05742	0.34714	0.05630	0.34725	0.06702	0.36321	0.06413
**FgMDMRS**	0.32643	0.05558	0.34790	0.05584	0.33204	0.05715	0.36117	0.06270
**FgMDMRU**	0.32783	0.06074	0.34509	0.05048	0.33592	0.06444	0.34747	0.06106

## Data Availability

The datasets analyzed for this study can be found in the EEG motor movement/imagery dataset: https://physionet.org/content/eegmmidb/1.0.0/ (1 March 2023).

## Supplementary Materials

The following supporting information can be downloaded at: https://www.mdpi.com/article/10.3390/s23115051/s1, Supplementary Figure S1: Experimental paradigm: Each experiment run consists of a 4-second relax trial followed by a 4-second motor imagery (or motor execution) trial. Each run is composed of 15 pairs of relax/action trials making a 2-minute recording. The subject starts the corresponding action based on a cue; Supplementary Figure S2: Classifiers’ performance: The accuracy of all classifiers under different scenarios. The errors bars represent one standard deviation; Supplementary Figure S3: Subject-wise MDM classifier performance: MDM classifier performance when classifying ME vs MI tasks for all subjects using 29 electrodes and 64 electrodes; Supplementary Figure S4: MDM Schematic Diagram; Supplementary Figure S5: MDMS Schematic Diagram; Supplementary Figure S6: MDMU Schematic Diagram; Supplementary Figure S7: MDMR Schematic Diagram; Supplementary Figure S8: MDMRS Schematic Diagram; Supplementary Figure S9: MDMRU Schematic Diagram; Supplementary Figure S10: FgMDM Schematic Diagram; Supplementary Figure S11: FgMDMS Schematic Diagram; Supplementary Figure S12: FgMDMU; Supplementary Figure S13: FgMDMR Schematic Diagram; Supplementary Figure S14: FgMDMRS; Supplementary Figure S15: FgMDMRU Schematic Diagram; Supplementary Figure S16: The classification accuracy per class for MDM classifier under the different scenarios; Supplementary Figure S17: The classification accuracy per class for MDMS classifier under the different scenarios; Supplementary Figure S18: The classification accuracy per class for MDMU classifier under the different scenarios; Supplementary Figure S19: The classification accuracy per class for MDMR classifier under the different scenarios; Supplementary Figure S20: The classification accuracy per class for MDMRS classifier under the different scenarios.; Supplementary Figure S21: The classification accuracy per class for MDMRU classifier under the different scenarios; Supplementary Figure S22: The classification accuracy per class for FgMDM classifier under the different scenarios; Supplementary Figure S23: The classification accuracy per class for FgMDMS classifier under the different scenarios; Supplementary Figure S24: The classification accuracy per class for FgMDMU classifier under the different scenarios; Supplementary Figure S25: The classification accuracy per class for FgMDMR classifier under the different scenarios; Supplementary Figure S26: The classification accuracy per class for FgMDMRS classifier under the different scenarios; Supplementary Figure S27: The classification accuracy per class for FgMDMU classifier under the different scenarios; Supplementary Figure S28: MDM Classifier Confusion matrices; Supplementary Figure S29: MDMS Classifier Confusion matrices; Supplementary Figure S30: MDMU Classifier Confusion matrices; Supplementary Figure S31: MDMR Classifier Confusion matrices; Supplementary Figure S32: MDMRS Classifier Confusion matrices; Supplementary Figure S33: MDMRU Classifier Confusion matrices; Supplementary Figure S34: FgMDM Classifier Confusion matrices; Supplementary Figure S35: FgMDMS Classifier Confusion matrices; Supplementary Figure S36: FgMDMU Classifier Confusion matrices; Supplementary Figure S37: FgMDMR Classifier Confusion matrices; Supplementary Figure S38: FgMDMRS Classifier Confusion matrices; Supplementary Figure S39: FgMDMRU Classifier Confusion matrices; Supplementary Table S1: Riemannian-geometry decoding algorithms (RGDAs) in brain–computer interface (BCI) classification: current literature landscape; Supplementary Table S2: Tasks, runs, and annotations.; Supplementary Table S3: Pros and Cons of different adaptation strategies; Supplementary Table S4: Pairwise comparison of the classifiers when classifying ME Tasks using 29 electrodes; Supplementary Table S5: Pairwise comparison of the classifiers when classifying MI Tasks using 29 electrodes; Supplementary Table S6: Pairwise comparison of the classifiers when classifying ME Tasks using 64 electrodes; Supplementary Table S7: Pairwise comparison of the classifiers when classifying MI Tasks using 64 electrodes.; Supplementary Table S8: Pairwise comparison of different classifier adaptation strategies: ME vs. MI when using 29 electrodes.; Supplementary Table S9: Pairwise comparison of different classifier adaptation strategies: ME vs. MI when using 64 electrodes; Supplementary Table S10: Pairwise comparison of different classifier adaptation strategies: 29 electrodes vs. 64 electrodes for ME trials; Supplementary Table S11: Pairwise comparison for different classifier adaptation strategies: 29 electrodes vs. 64 electrodes for MI trials.

## Abbreviations

The following abbreviations are used in this manuscript:

BCI	Brain–computer Interface
BL	Bilateral
BPF	Band-pass filter
CM	Covariance matrix
CSP	Common spatial pattern
CV	Cross validation
DTF	Directed transfer function
EDF+	European Data Format ’plus’ files
EEG	Electroencephalography or Electroencephalogram
ERD	Event-related desynchronization
ERS	Event-related synchronization
FGMDM	Geodesic minimum distance to Riemannian mean
FgMDMRS	Supervised FgMDMR
FGMDMRU	Unsupervised FgMDMR
ITR	Information Transfer Rate
MDM	Minimum distance to Riemannian mean
MDMR	Rebias MDM
MDMRS	Supervised MDMR
MDMRU	Unsupervised MDMR
MDMS	Supervised MDM
MDMU	Unsupervised MDM
ME	Motor execution
MI	Motor imagery
MLP	Multi-layer perceptron
MRC-MLP	Multiple Riemannian covariance multilayer perception
NF	Notch filtering
R-MDRM	Regularized minimum distance to the Riemannian mean
RCSP	Regularized CSP
RG	Riemannian geometry
RGDA	Riemannian geometry decoding algorithm
RM ANOVA	Repeated measures Analysis of Variance
RSP	Riemannian spatial pattern
SCM	Spatial CM
SMR	Sensorimotor Rhythms
SNR	Signal-to-noise ratio
SPD	Symmetric positive definite
SPSS	Statistical Package for Social Sciences
SSDT	Subject-specific decision tree
SVM	Support vector machine
UL	Unilateral
WFDB	WaveForm DataBase
