# Supplementary material for: Decoding Multi-Class Motor Imagery and Motor Execution Tasks Using Riemannian Geometry Algorithms on Large EEG Datasets
Source: Sensors (Basel). 2023 May 25;23(11):5051. doi: 10.3390/s23115051 (PMC10255410; doi:10.3390/s23115051)
Supplement: Supplementary file 1 [file sensors-23-05051-s001.zip › sensors-2310030-supplementary.pdf]

SUPPLEMENTARY INFORMATION FOR

Decoding Multi-class Motor Imagery and Motor Execution Tasks Using Riemannian Geometry Algorithms on Large EEG Datasets

Zaid Shuqfa, Abdelkader Nasreddine Belkacem (Senior Member, IEEE) and Abderrahmane Lakas (Senior Member, IEEE)

College of Information Technology, UAE University, Al Ain 15551, United Arab Emirates

Contents:

Table of Contents

Current literature: Comparative Table 2
Dataset Description and Experimental Paradigm 5
Comparison of Different RGDA Adaptation Strategies 7
Classifiers Performance 8
Schematic Diagrams of Classifiers Adaptation Strategies 10
Classification Accuracy Per Class 14
Confusion Matrices 21
Simple Main Effect and Pairwise Comparison Data Sheets 27

Current literature: Comparative Table

Supplementary Table S1: Riemannian-geometry decoding algorithms (RGDAs) in brain–computer interface (BCI) classification: current literature landscape
The refernece number is as per the manuscript. When refering to a section the section is in the manuscript.

Paper	Dataset	Subjects	Channels	Sampling [Hz]	Sessions	Tasks	Trials	Classes	Epoching	Pre-processing	Feature Extraction	Calibration /Evaluation	Measure	Classifier	Performance
[14]	BCI comp. IV: dataset IIa	9	22		2	MI: L Hand, R Hand, lounge, Foot	4s 72 trial /task	4	0.5 s to 3.5 s	8 Hz to 30 Hz 5th BwBPF	SBE CM	Session 1 to Session 2	Acc.	MDM	0.628
													Acc.	MDMS	0.536
													Acc.	MDMU	0.648
													Acc.	MDMR	0.647
													Acc.	MDMRS	0.668
													Acc.	FgMDM	0.689
													Acc.	FgMDMS	0.667
													Acc.	FgMDMU	0.743
													Acc.	FgMDMR	0.711
													Acc.	FgMDMRS	0.734
	In-house	18	30		6	Mental[a]	8s 15 trial /task /run	3		8 Hz to 30 Hz 50th FIR BPF		Session 1 to Sessions 2-6	Acc.	MDM	0.560
													Acc.	MDMS	0.385
													Acc.	MDMU	0.583
													Acc.	MDMR	0.661
													Acc.	MDMRS	0.697
													Acc.	FgMDM	0.585
													Acc.	FgMDMS	0.576
													Acc.	FgMDMU	0.727
													Acc.	FgMDMR	0.703
													Acc.	FgMDMRS	0.762
[13]	BCI comp. IV: dataset IIa	9	22 + 3 EOG		2	MI: L Hand, R Hand, lounge, Foot	72 trial /task = 288	4	0.5 s to 2.5 s	8 Hz to 30 Hz 5th BwBPF		10-fold CV	Acc. (OVR)	SSDT with FGMDM	L = 0.820
															R = 0.813
															F = 0.815
															T = 0.840
													Kappa Coeff.		0.589
													Acc. (OVO)	SSDT with FGMDM	L/R = 0.794
															L/F = 0.871
															L/T = 0.865
															R/F = 0.868
															R/T = 0.870
															F/T = 0.820
													Kappa Coeff.		0.607
	BCI comp. III: dataset IIIa	3	64	250		MI: L Hand, R Hand, lounge, Foot		4			SJGDA	5-fold CV	Acc.	SSDT-KNN	0.828
	In-house	7	14	128		MI: Shoulder (flexion, extension, abduction)	5s 20 trials	3			SJGDA	5-fold CV	Acc.	SSDT-KNN	≈ 0.850

Continued on next page

[a]Left-hand MI; mental imagery: rotation of a 3D geometric figure, and mental subtraction of a two-digit number from a three-digit number

Paper	Dataset	Subjects	Channels	Sampling [Hz]	Sessions	Tasks	Trials	Classes	Epoching	Pre-processing	Feature Selection	Calibration /Evaluation	Measure	Classifier	Performance
**[24]**	BCI comp. III: dataset Iva	5	118	100		MI: R Hand Foot	140 trial /task = 280	2	0.5 s to 2.5 s	7 Hz to 30 Hz 5th BwBPF	Sample CM	Uneven number of trials[b]	Acc.	R-MDRM	0.872
													Kappa Coeff.		0.740
	BCI comp. III: dataset IIIa	3	60	250		MI: L Hand, R Hand, lounge, Foot		2	0.5 s to 2.5 s	7 Hz to 30 Hz 5th BwBPF	Sample CM		Acc.		0.909
	BCI comp. IV: dataset IIa	9	22	250		MI: L Hand, R Hand, lounge, Foot	72 trial /task 288	2	0.5 s to 2.5 s		Sample CM		Acc.		0.810
[29]	Clinical dataset	1	64 ECoG	586	4[c]	MI[d]	200	12	0.0 s to 1.0 s	60 Hz to 130 Hz BPF	Spatial CM	10-fold CV	CP	MDM	0.856
														tsSVM	0.885
[32]	BCI comp. IV: dataset IIa	9	22 + 3 EOG	250	2	MI: L Hand, R Hand, lounge, Foot	72 trial /task 288	4	2.5 s to 6.0 s	0.5 Hz to 100 Hz and 50Hz NF	MRC	Session 1 to Session 2	Acc.	MLP	0.761
[5]	BCI comp. IV: dataset I	7				MI: L Hand, R Hand	100 trial /task 200	2		8 Hz to 30 Hz 5th BPF and 50 Hz NF	Sample CM		Acc.	MDM	0.770[e]
	BCI lab. Shandong Jianzhu University	4				MI: L Hand, R Hand, lounge, Foot	10s 150 trials	3		8 Hz to 30 Hz BPF and 50 Hz NF	Sample CM		Acc.[f]		0.800
[26]	BCI comp. IV: dataset IIa	9	22			MI: L Hand Foot	144 trial /task	4	0.5 s to 2.5 s	8 Hz to 30 Hz 5th BwBPF	Spatial CM	30-fold CV	Acc.	MDM	0.632
														TSLDA	0.702

Continued on next page

[b] Uneven number of trials among subjects

[c] 2 right arm, 2 left, 2 right finger, and 2 left (20 minute each)

[d] Left and right arm (elbow flexion, wrist extension, and hand grasping), and left and right hand (finger flexion of the thumb, index finger, and middle finger) ‘Arm: 2s 50 trials/task = 150 Finger: 1s 8[illegible] [illegible]ials/(thumb and index task) + 40 trials/middle finger task = 200

[e] Four subjects only were included

[f] The average of binary classification accuracy (LH-RH, LH-F, RH-F)

Paper	Dataset	Sub-jects	Cha-nnels	Sampling [Hz]	Sess-ions	Tasks	Trials	Classes	Epoch-ing	Pre-proce-ssing	Feature Selection	Calibration /Evaluation	Measure	Classifier	Perform-ance
**[23]**	BCI comp. III: dataset IVa	5	9			MI: R Hand R Foot	280 trials	2		10 Hz to 30 Hz BPF	SPD	10-fold CV	ER	RDC	0.191
														FRDC	0.148
This paper[g]	physionet EEG Mo-tor Movement /Imagery	103	64	160	1	ME: L Hand, R Hand, Both Hands, Both Feet	4s 21 to 24 trial /task	8	0.0 s to 4.0 s	8 Hz to 30 Hz 5th BwBPF	Sample CM	10-fold CV	Acc.	MDM	0.815
			29										Acc.	MDM	0.765
			64			MI: L Hand, R Hand, Both Hands, Both Feet							Acc.	MDM	0.764
			29										Acc.	MDM	0.721

Table Acronyms and Abbreviations:

Acc:	Mean Accuracy (%)
BPF:	Bandpass Filter
BwBPF:	Butterworth Bandpass Filter
Coeff:	Coefficient
CP:	Classification Performance
FGMDM:	Filter Geodesic Minimum Distance to Riemannian Mean
FIR:	Finite Impulse Response
FRDC:	Simple Riemannian Distance Classification, Filtered Version
MLP:	Multi-layer Perceptron
MRC:	Multiple Riemannian Covariance
NF:	Notch Filtering
OVO:	One-vs-One
OVR:	One-vs-Rest
RDC:	Simple Riemannian Distance Classification
SBE:	Shrinkage-based Estimator
SPD:	Symmetric Positive Definite
SSDT:	Subject-specific Decision Tree
TSLDA:	Tangent Space Linear Discriminant Analysis
tsSVM:	Tangent-space Support Vector Machine

[g] For the rest of results, refer to the paper

Dataset Description and Experimental Paradigm

The dataset EEG Motor Movement/Imagery Dataset v1.0.0 consists of 1500 EEG recordings taken from 109 volunteered subjects. Each subject has 26-minute EEG recordings in European Data Format 'plus' files (EDF+). The run files are structured in folders, each of which is named using the subject identifier; e.g., `S015` for subject number 15. The name of the run file concatenates the subject identifier and the run number, e.g., `S099R11.edf` is the name of the file containing run 11 of subject 99.

The data were obtained using a general-purpose BCI system (BCI2000). The signals were sampled at 160 Hz from 64 electrodes as per the international 10-10 system excluding electrodes `Nz, F9, F10, FT9, FT10, A1, A2, TP9, TP10, P9,and P10`. Each volunteer was subjected to 14 EEG-recording runs as described in the table below, where the first two were baseline runs and are not used for classification in the present study.

Supplementary Table S2: Tasks, runs, and annotations.

Task	Description	UL or BL	ME or MI	Runs	Annotations			Duration for each run	Used in the classification
					T0	T1	T2		
**Baseline 1**	Keep eyes open	-	-	1	-	-	-	1 minute	No
**Baseline 2**	Keep eyes closed	-	-	2	-	-	-	1 minute	No
**Task 1**	Open and close right/left fist	UL	ME	3, 7, 11	Relax	Left fist	Right fist	2 minutes	Yes
**Task 2**	Imagine opening and closing right/left fist	UL	MI	4, 8, 12	Relax	Left fist	Right fist	2 minutes	Yes
**Task 3**	Open and close both fists/feet	BL	ME	5, 9, 13	Relax	Both fists	Both feet	2 minutes	Yes
**Task 4**	Imagine opening and closing both fists/feet	BL	MI	6, 10, 14	Relax	Both fists	Both feet	2 minutes	Yes

UL: Unilateral
BL: Bilateral
ME: Motor Execution
MI: Motor Imagery

During each run, when the subject saw a target on the screen, they corresponded to its position depending on the task under the experiment. Supplementary Figure S1 shows the experimental procedure. In an ME run (Task 1) for example, if the target appeared on either side of the screen (horizontally), then the subject opened and closed the corresponding fist until the target disappeared; otherwise, for MI runs (Task 2), they imagined opening and closing the corresponding fist until the target disappeared. The same applied to Tasks 3 and 4, except that they were Bilateral (BL) tasks, i.e., the subject opened and closed (or imagined opening and closing) both fists or feet when the target appeared on the top or bottom of the screen, respectively (see the figure below).

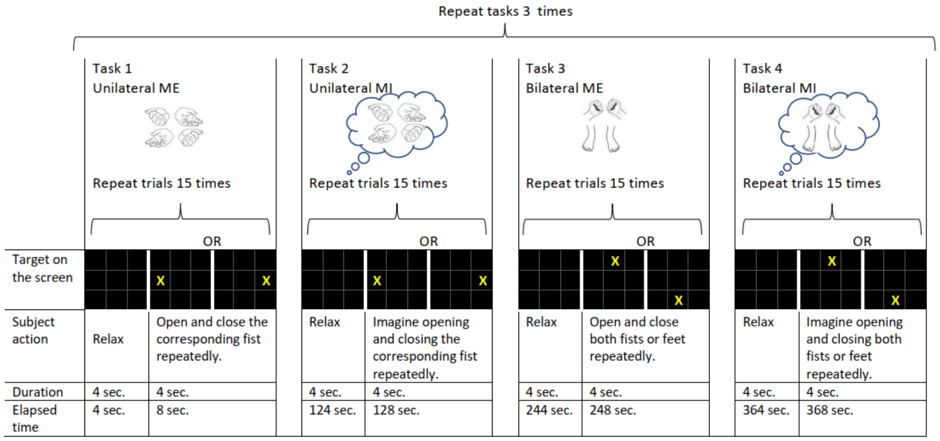

Supplementary Figure S1: Experimental paradigm: Each experiment run consists of a 4-second relax trial followed by a 4-second motor imagery (or motor execution) trial. Each run is composed of 15 pairs of relax/action trials making a 2-minute recording. The subject starts the corresponding action based on a cue.

Each trial was 4-second long and was preceded by a 4-second relax trial. The single run comprised 15 pairs of relax and execution/imagery trials, creating a two-minute run containing 30 trials; each run was saved as an EDF+ file. Three trials annotated as T0, T1, and T2 are in each run, where the annotation differs according to the task under consideration (see Supplementary Table S2).

Comparison of Different RGDA Adaptation Strategies

Supplementary Table S3: Pros and Cons of different adaptation strategies

Method	Pros	Cons
**Baseline**	Straight forward and simple concept<br>Relatively less computational complexity when compared with the adaptation strategies.<br>Provides better performance when data samples are limited [45].	Static, does not adapt with new trials, hence does not accommodate with intersession and inter subject variability [40]
**Supervised**	The parameters of the classifier update gradually while testing to deal with variability [40]<br>It can be calibrated initially with fewer data samples and the fine-tuning goes as the classifier is used online (or while testing)<br>It's the best adaptation strategy in terms of performance	Data labels are required during testing [14] which is not realistic assumption in real applications unless there is a user feedback mechanism [40]<br>It requires more data samples to train properly [45]
**Unsupervised**	The parameters of the classifier update gradually while testing to deal with variability [40]<br>It can be calibrated initially with fewer data samples and the fine-tuning goes as the classifier is used online (or while testing)<br>The class prototype updates based on the predicted label which is a realistic assumption [45]	It adapts depending on the predicted label [45], which may be an inaccurate prediction.<br>In comparison to the static classifier, it requires more data samples to train properly [45]<br>Its more difficult compared to the supervised ones [46]
**Rebiase**	Does not modify the classifier or its parameters, instead it shift the output to minimize the error. [14]	In comparison to the static classifier, it requires more data samples to train properly [45]<br>The subject BCI-literacy affects its performance, a lot [45].

Classifiers Performance

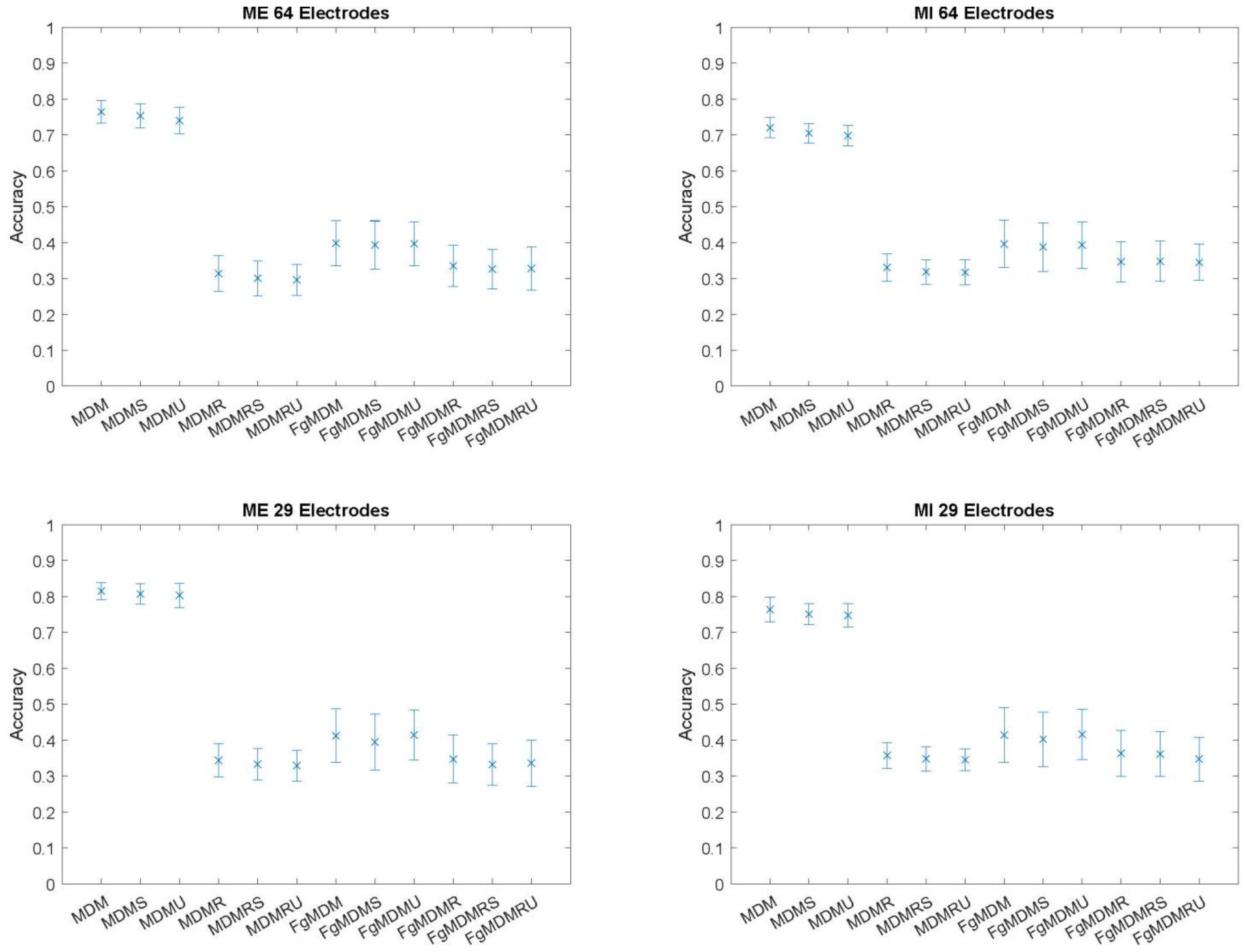

Supplementary Figure S2: Classifiers' performance: The accuracy of all classifiers under different scenarios. The errors bars represent one standard deviation

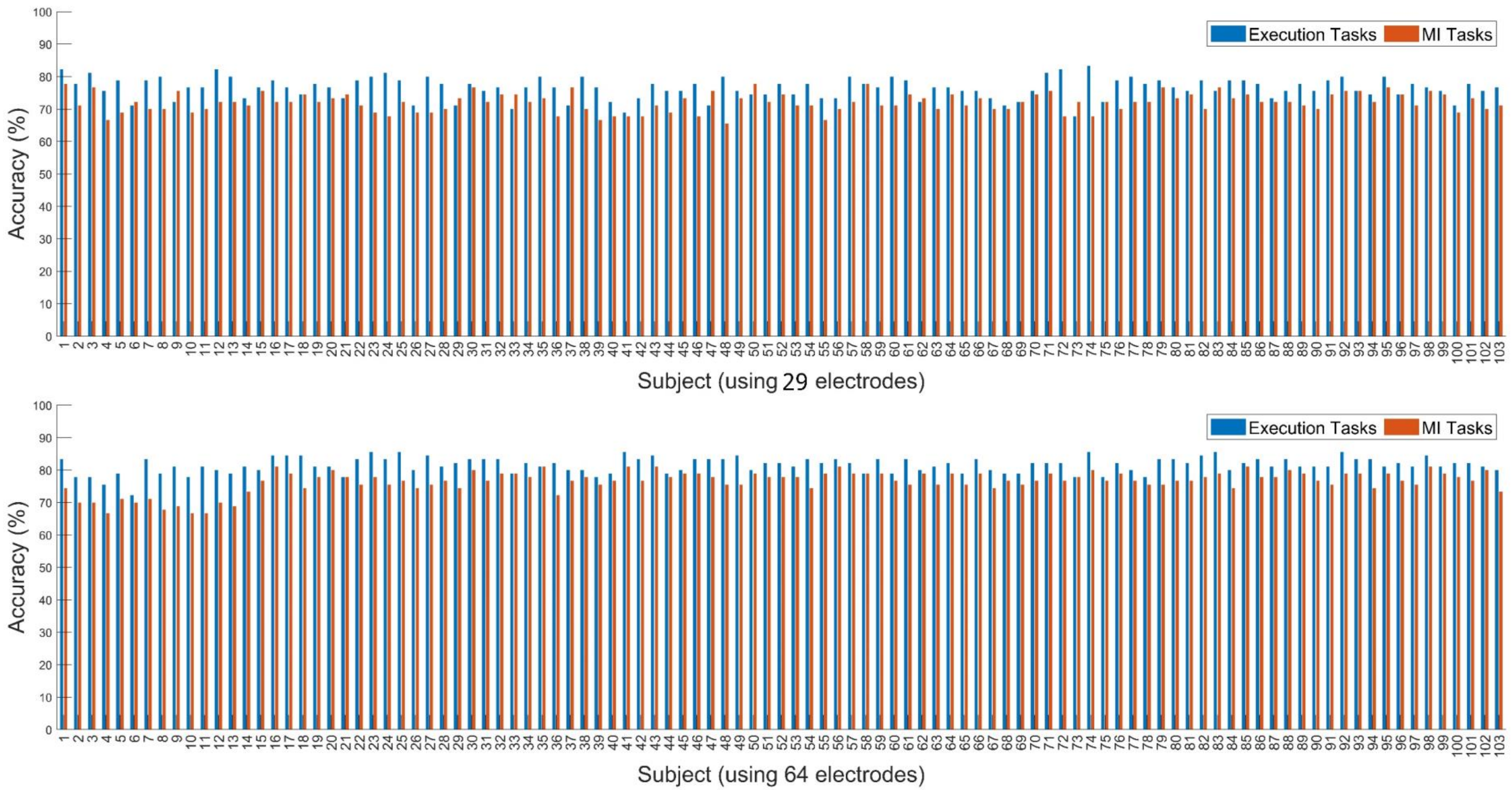

Supplementary Figure S3: Subject-wise MDM classifier performance: MDM classifier performance when classifying ME vs MI tasks for all subjects using 29 electrodes and 64 electrodes.

Schematic Diagrams of Classifiers Adaptation Strategies

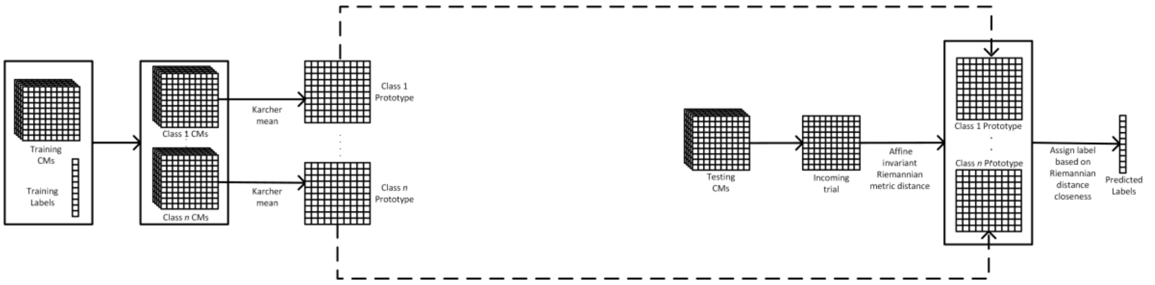

(a) Calibration/Testing (b) Evaluation/Testing

Supplementary Figure S4: MDM Schematic Diagram

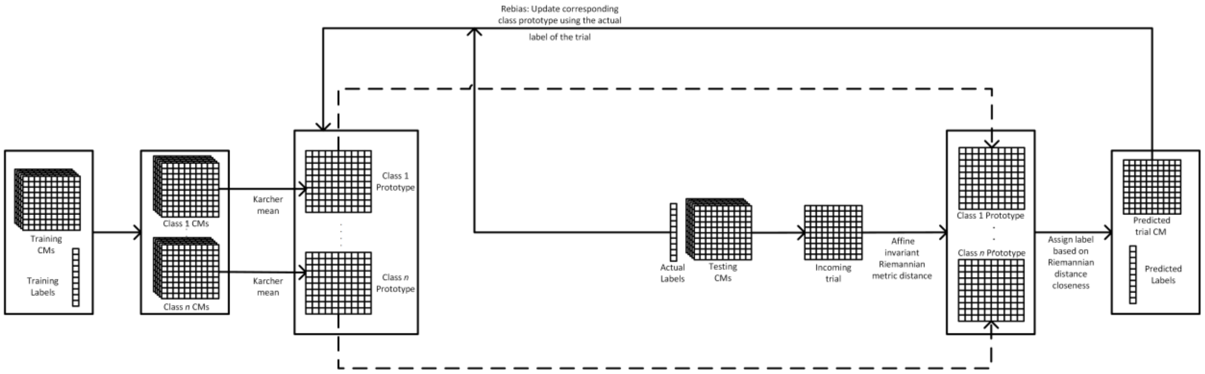

(a) Calibration/Testing (b) Evaluation/Testing

Supplementary Figure S5: MDMS Schematic Diagram

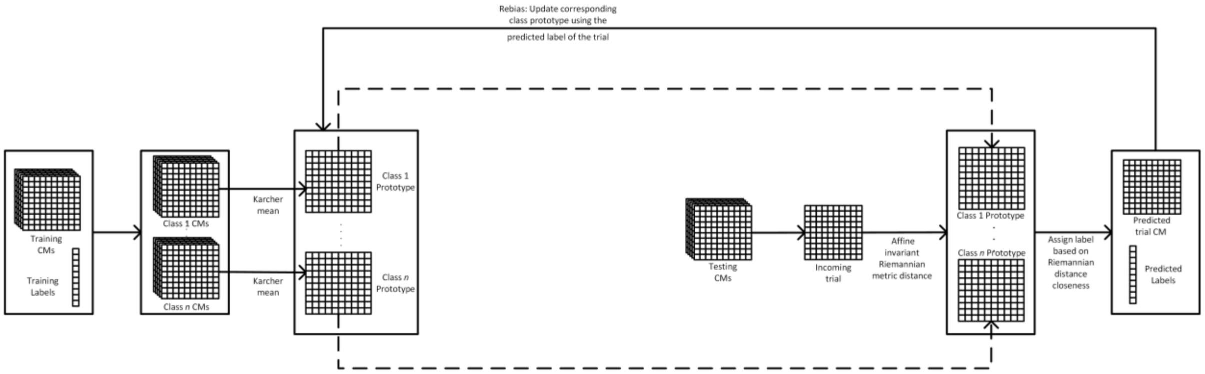

(a) Calibration/Testing (b) Evaluation/Testing

Supplementary Figure S6: MDMU Schematic Diagram

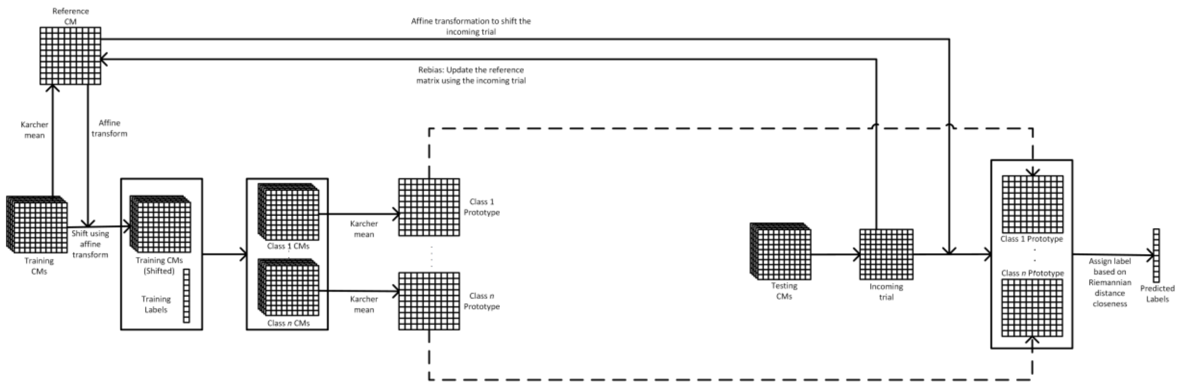

(a) Calibration/Testing (b) Evaluation/Testing

Supplementary Figure S7: : MDMR Schematic Diagram

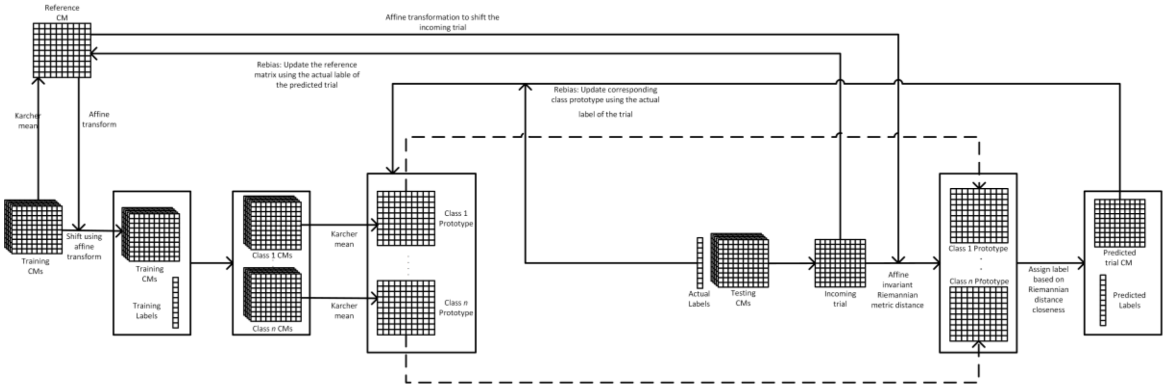

(a) Calibration/Testing (b) Evaluation/Testing

Supplementary Figure S8: MDMRS Schematic Diagram

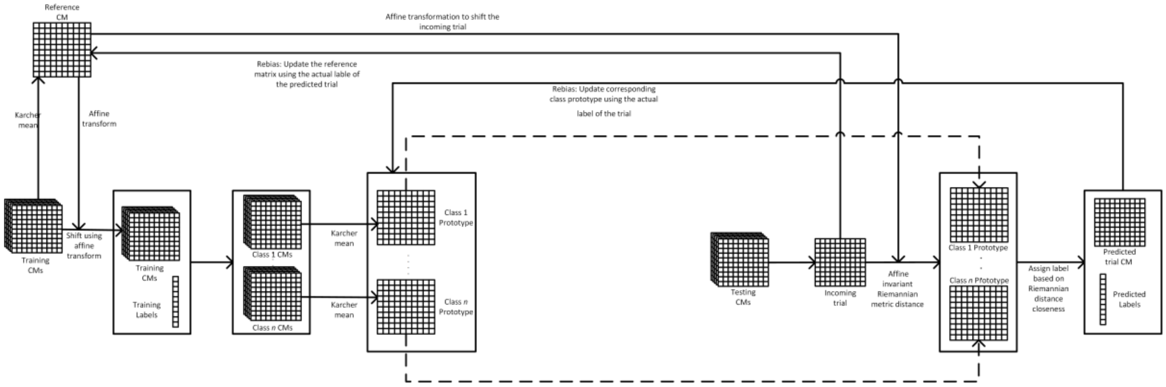

(a) Calibration/Testing (b) Evaluation/Testing

Supplementary Figure S9: MDMRU Schematic Diagram

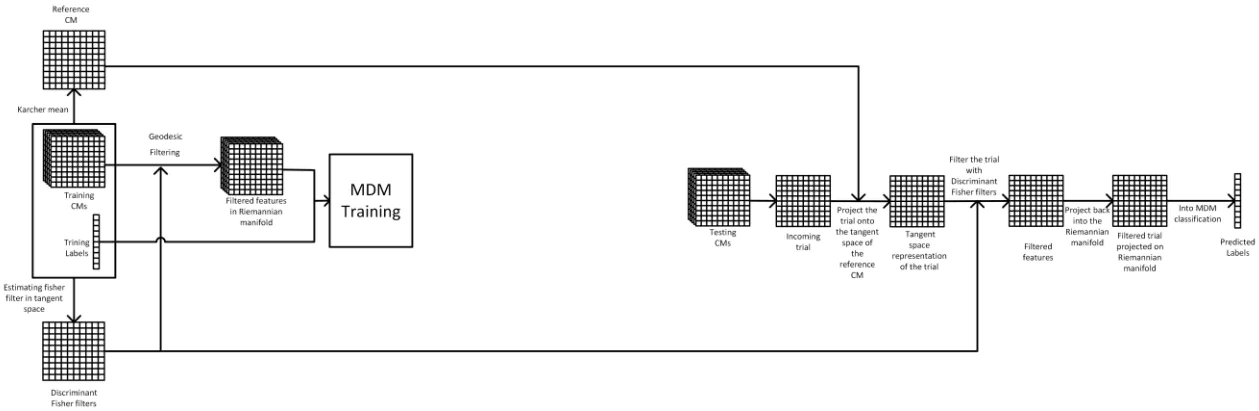

(a) Calibration/Testing (b) Evaluation/Testing

Supplementary Figure S10: FgMDM Schematic Diagram

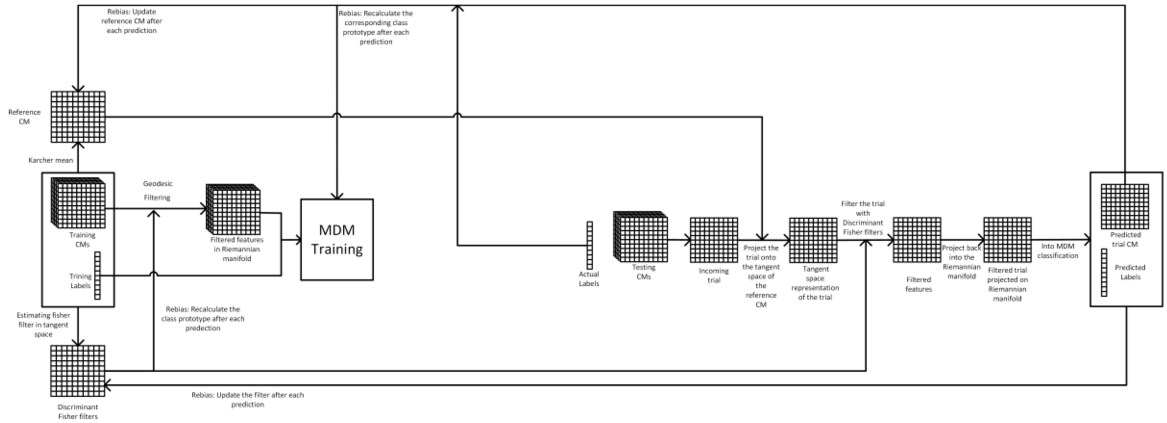

(a) Calibration/Testing (b) Evaluation/Testing

Supplementary Figure S11: FgMDMS Schematic Diagram

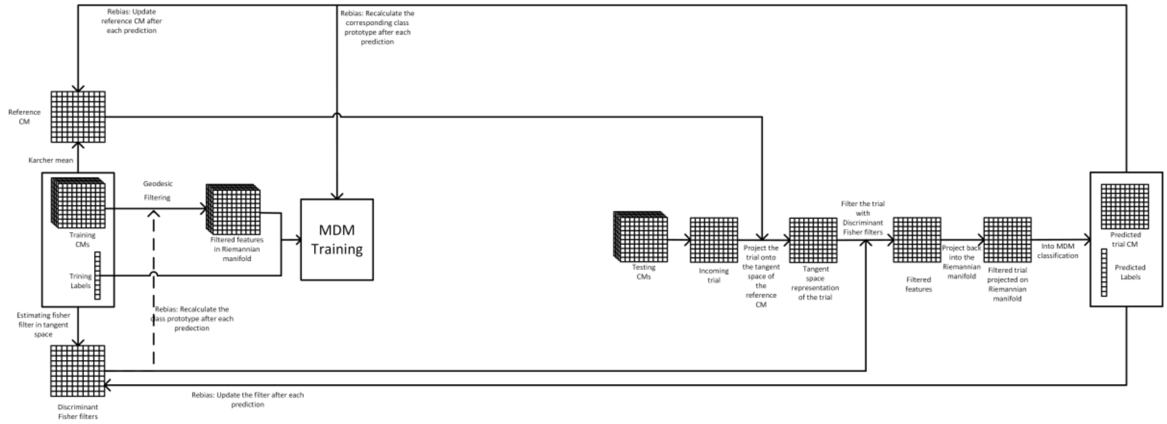

(a) Calibration/Testing (b) Evaluation/Testing

Supplementary Figure S12: FgMDMU

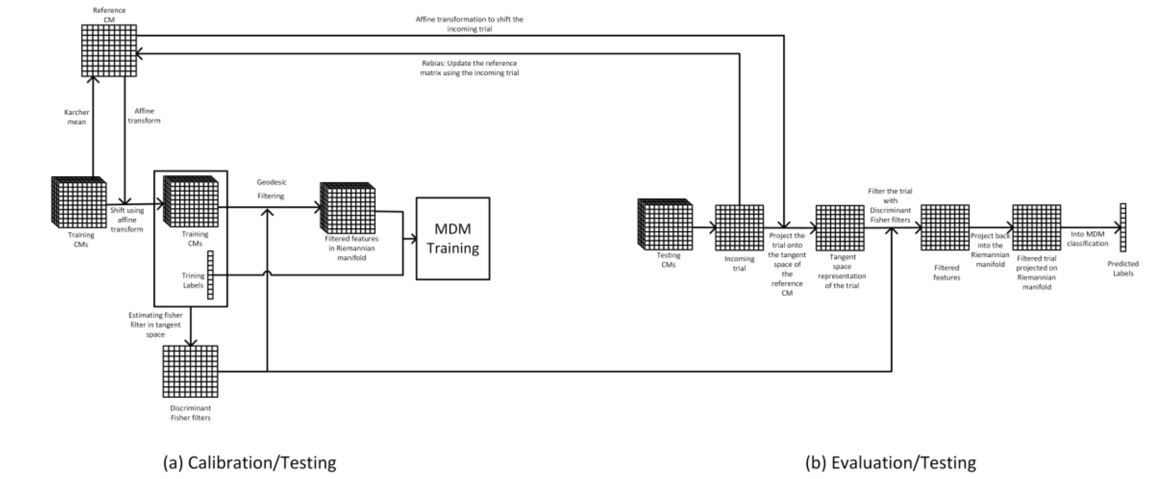

Supplementary Figure S13: FgMDMR Schematic Diagram

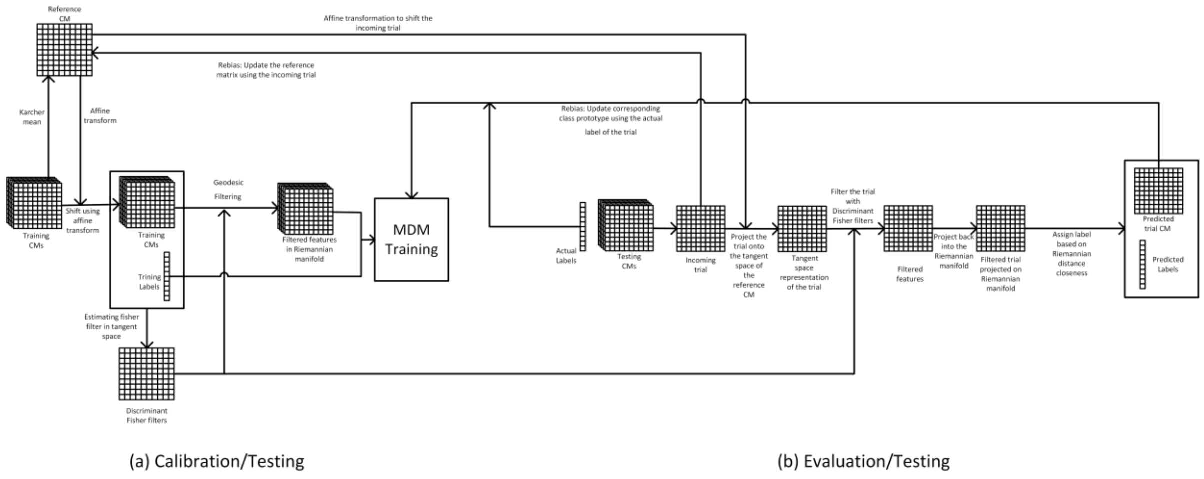

Supplementary Figure S14: FgMDMRS

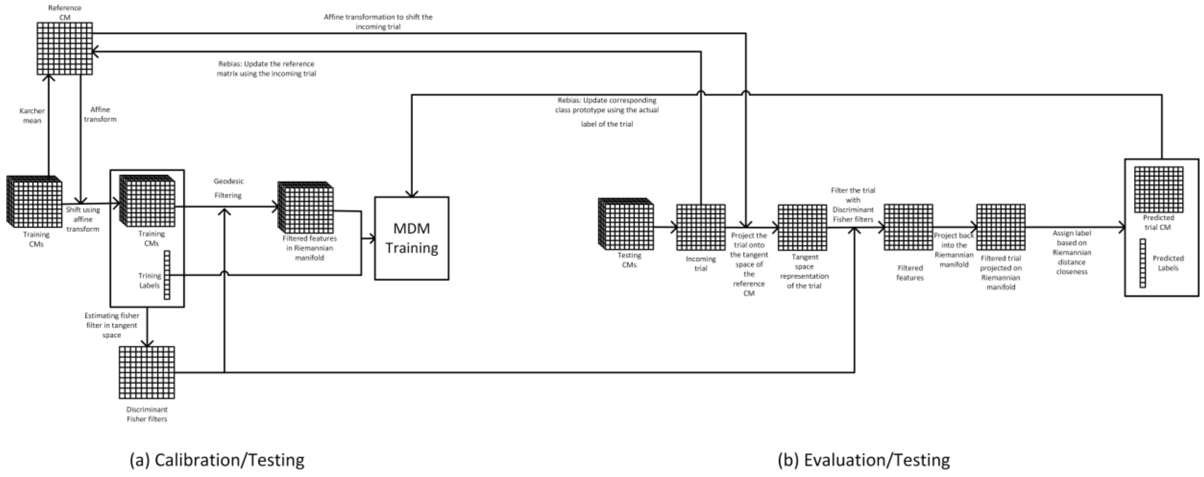

Supplementary Figure S15: FgMDMRU Schematic Diagram

Classification Accuracy Per Class

The accuracy per class for each classifier are presented in the figures 1-12 below. Consider the number of subjects is $n = 103$ and the summation index is s for subject and the folds are $k = 10$ and the summation index is f, then the accuracy per class (CA) is calculated according to the following formula. Note that the average class accuracy (ACA) represents the mean accuracy of the four classes, and it is slightly different than the overall classifier performance where $m = 4$ is the number of classes. For the classifier performance, please refer to the paper.

$$CA = \frac{\sum_{s=1}^{n} \frac{\sum_{f=1}^{k} \frac{TP_f + TN_f}{TP_f + TN_f + FP_f + FN_f}}{k}}{n}$$

$$ACA = \frac{CA_{class1} + CA_{class2} + CA_{class3} + CA_{class4}}{m}$$

The error bars represent the $\pm$ one standard deviation (CSD) of all classifier's accuracy for one class at a time. The error bar of the average column represents the average of the standard deviations of the four classes (ASD) as follows:

$$CSD = \sqrt{\frac{\sum_{s}^{n}(CA_s - \mu)^2}{n}}$$

$$ASD = \frac{CSD_{class1} + CSD_{class2} + CSD_{class3} + CSD_{class4}}{m}$$

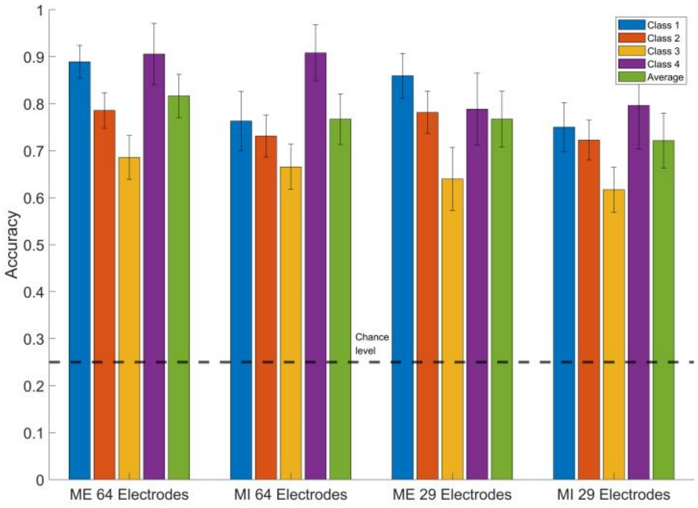

Supplementary Figure S16: The classification accuracy per class for MDM classifier under the different scenarios.

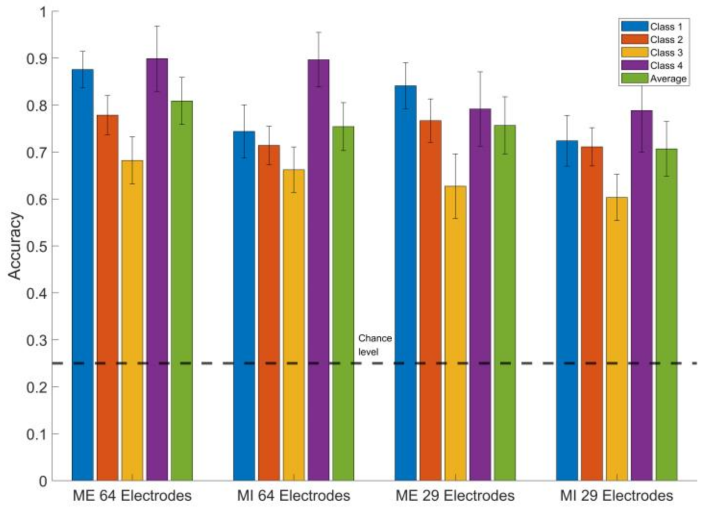

Supplementary Figure S17: The classification accuracy per class for MDMS classifier under the different scenarios.

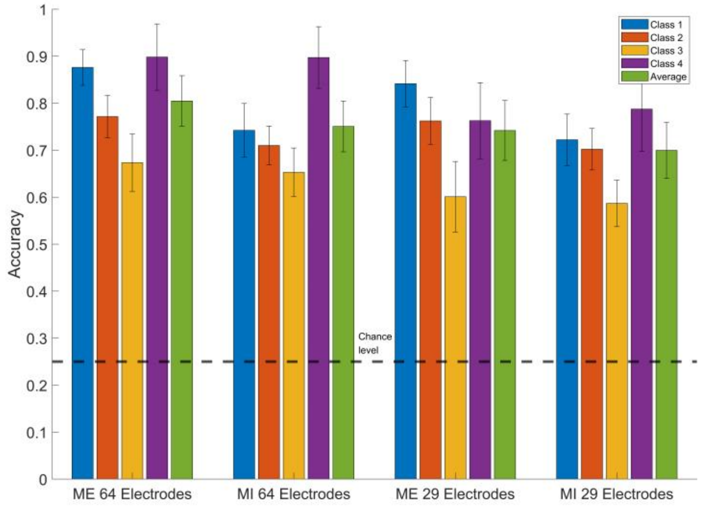

Supplementary Figure S18: The classification accuracy per class for MDMU classifier under the different scenarios.

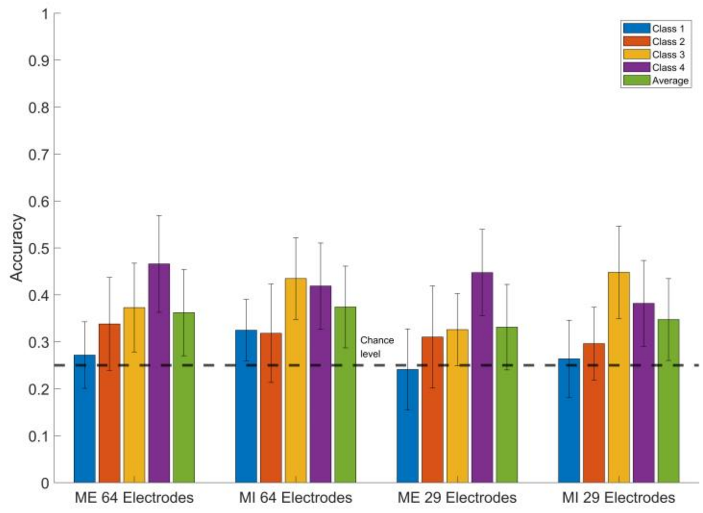

Supplementary Figure S19: The classification accuracy per class for MDMR classifier under the different scenarios.

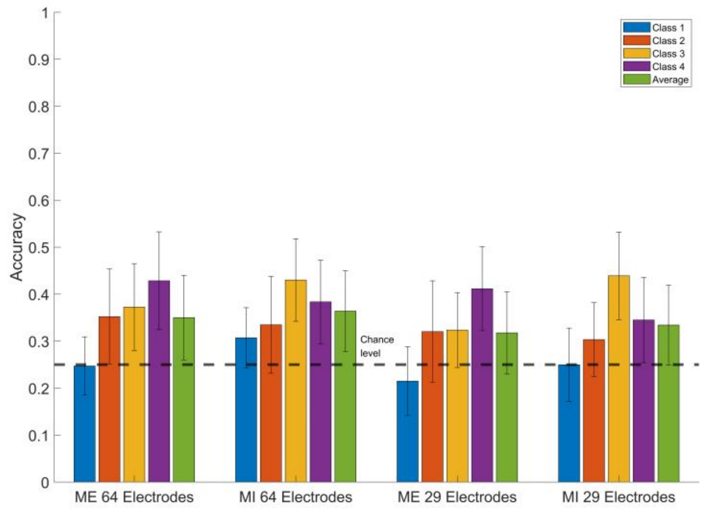

Supplementary Figure S20: The classification accuracy per class for MDMRS classifier under the different scenarios.

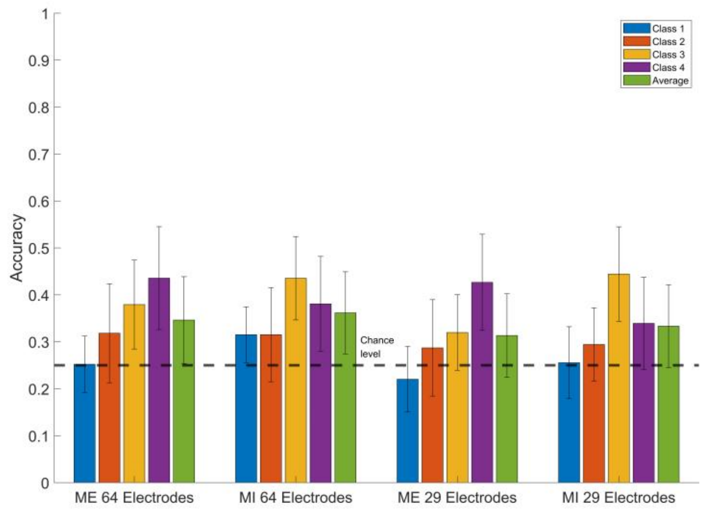

Supplementary Figure S21: The classification accuracy per class for MDMRU classifier under the different scenarios.

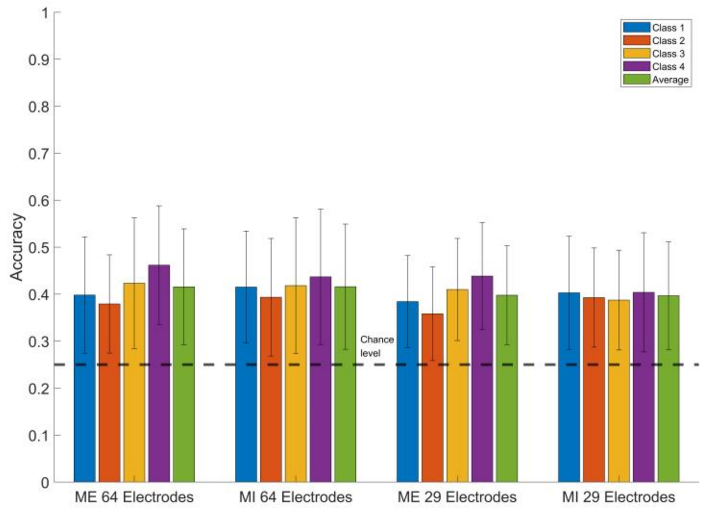

Supplementary Figure S22: The classification accuracy per class for FgMDM classifier under the different scenarios.

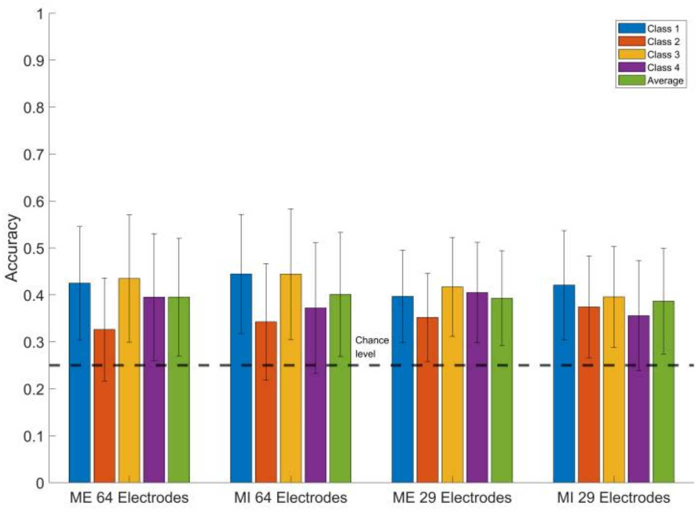

Supplementary Figure S23: The classification accuracy per class for FgMDMS classifier under the different scenarios.

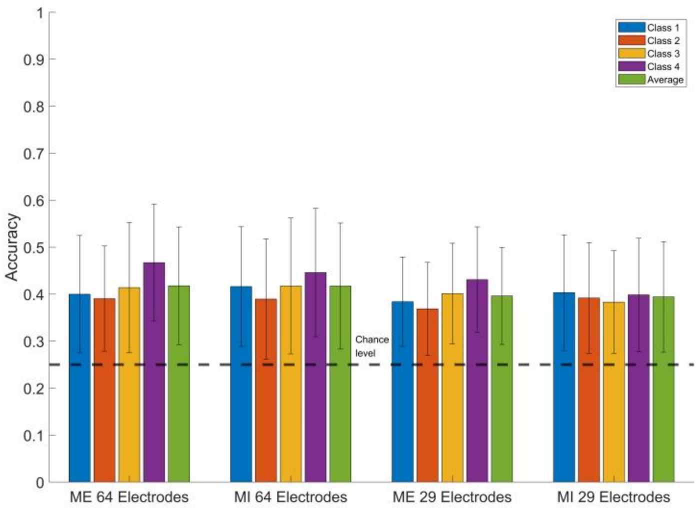

Supplementary Figure S24: The classification accuracy per class for FgMDMU classifier under the different scenarios.

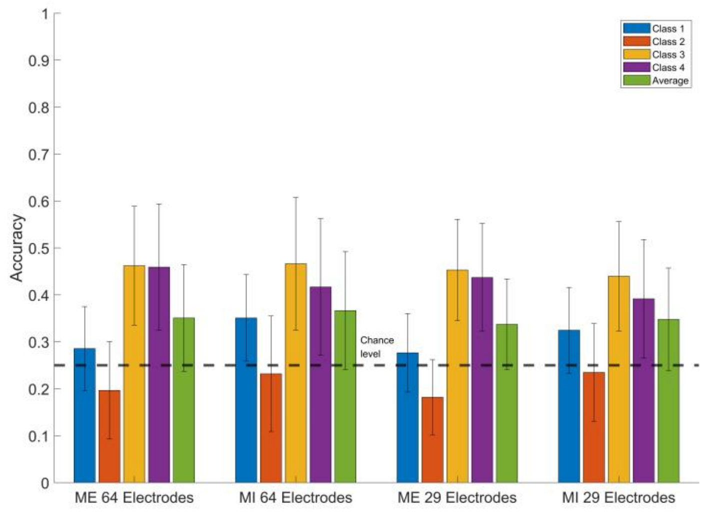

Supplementary Figure S25: The classification accuracy per class for FgMDMR classifier under the different scenarios.

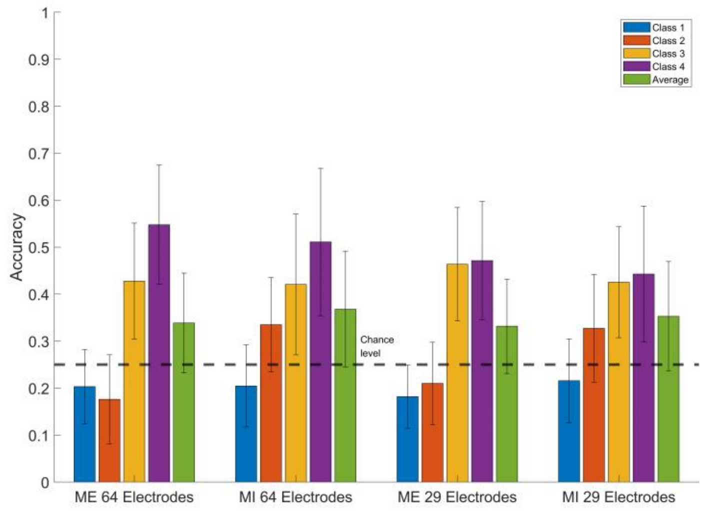

Supplementary Figure S26: The classification accuracy per class for FgMDMRS classifier under the different scenarios.

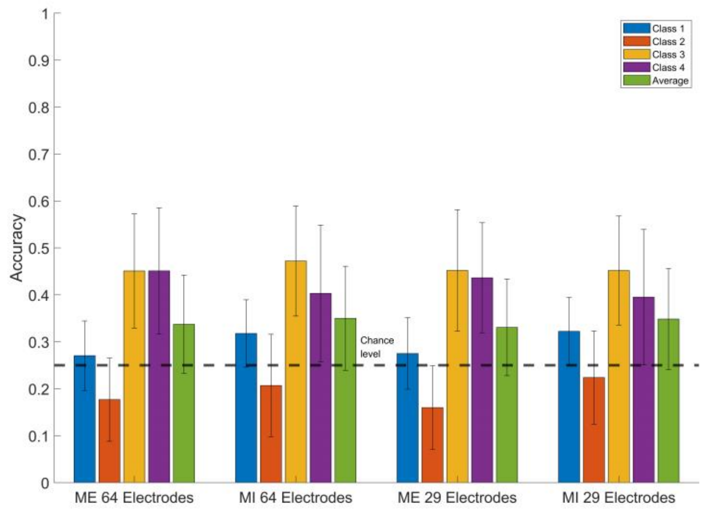

Supplementary Figure S27: The classification accuracy per class for FgMDMU classifier under the different scenarios.

Confusion Matrices

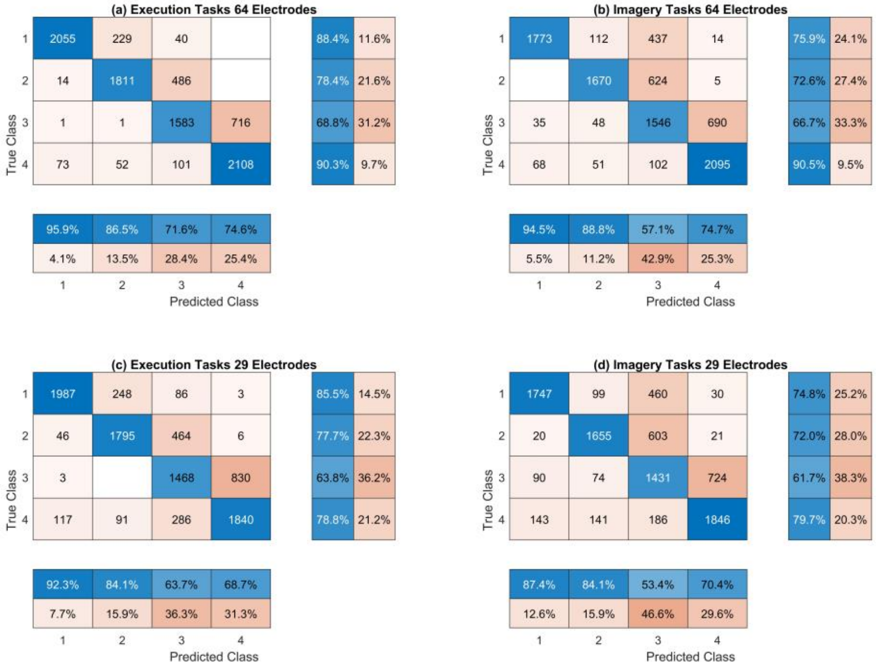

Supplementary Figure S28: MDM Classifier Confusion matrices

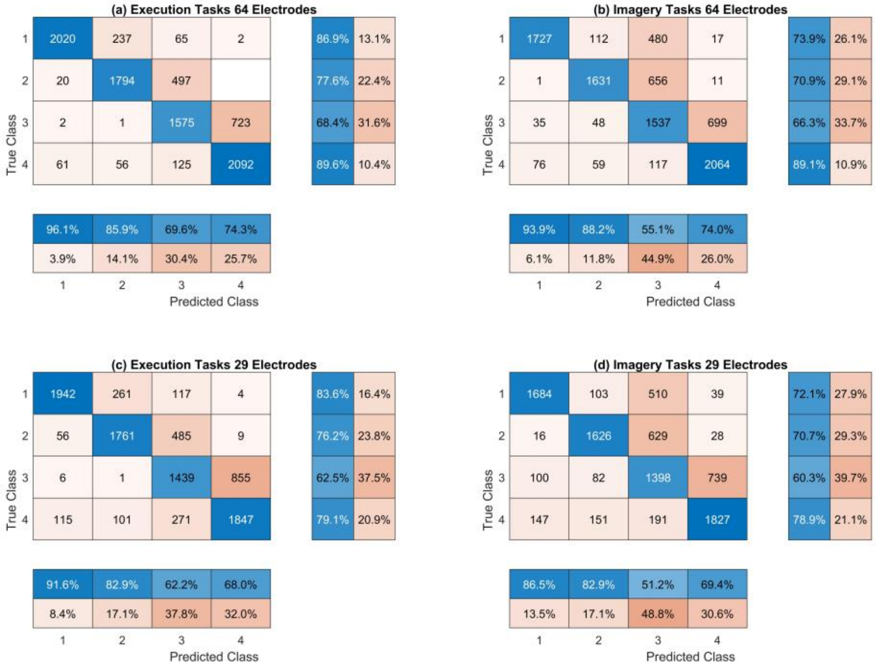

Supplementary Figure S29: MDMS Classifier Confusion matrices

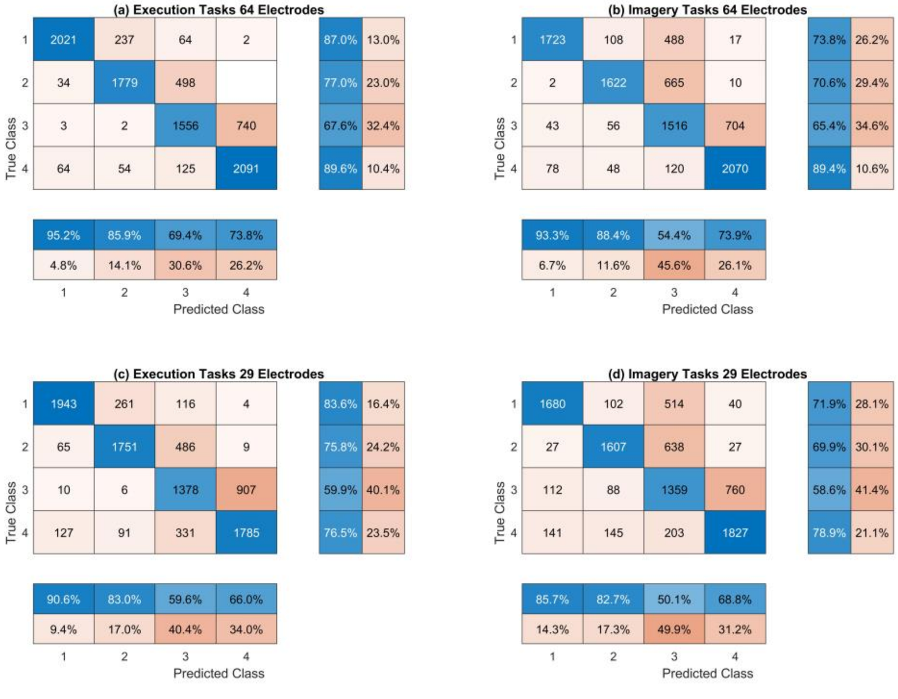

Supplementary Figure S30: MDMU Classifier Confusion matrices

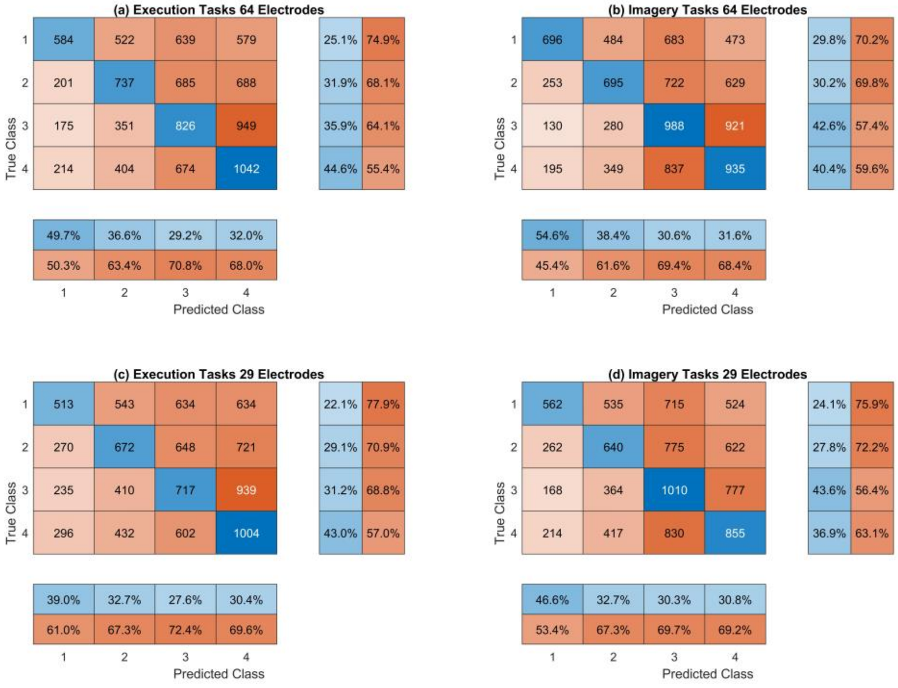

Supplementary Figure S31: MDMR Classifier Confusion matrices

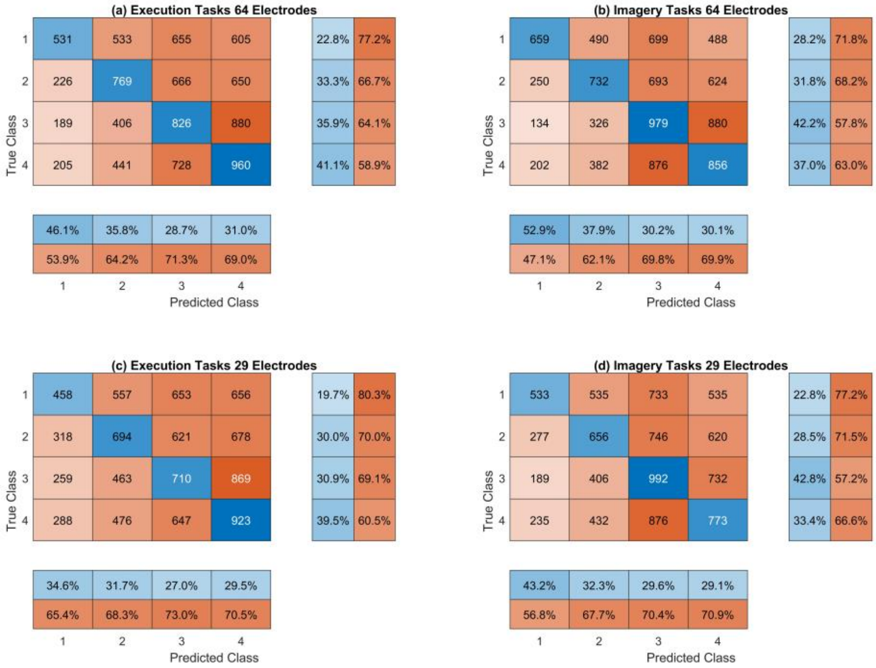

Supplementary Figure S32: MDMRS Classifier Confusion matrices

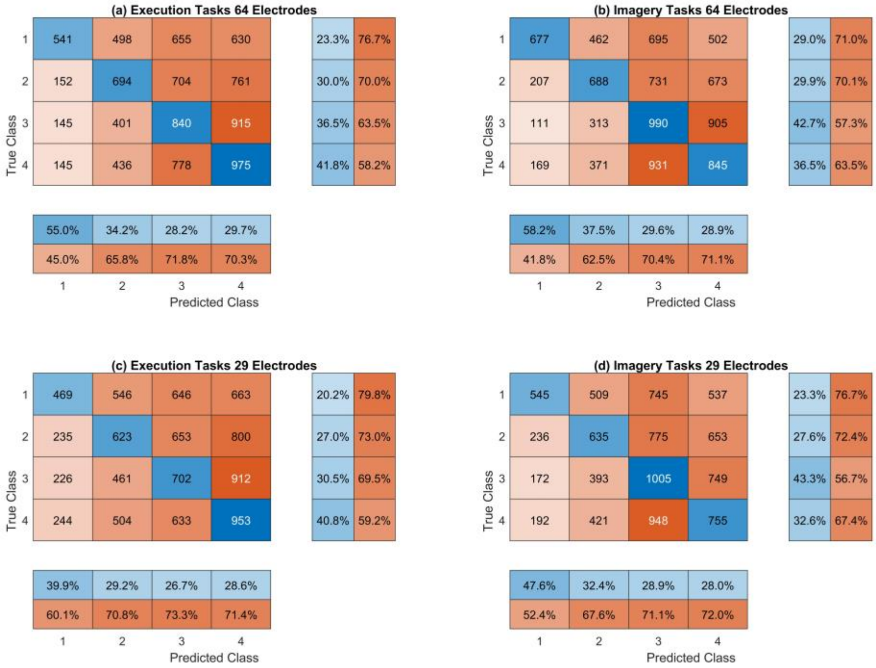

Supplementary Figure S33: MDMRU Classifier Confusion matrices

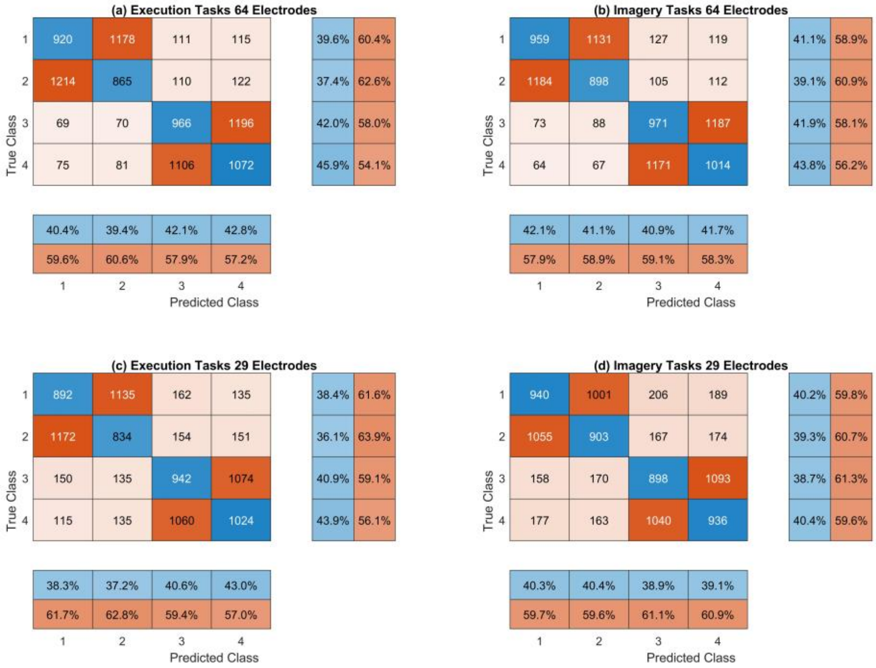

Supplementary Figure S34: FgMDM Classifier Confusion matrices

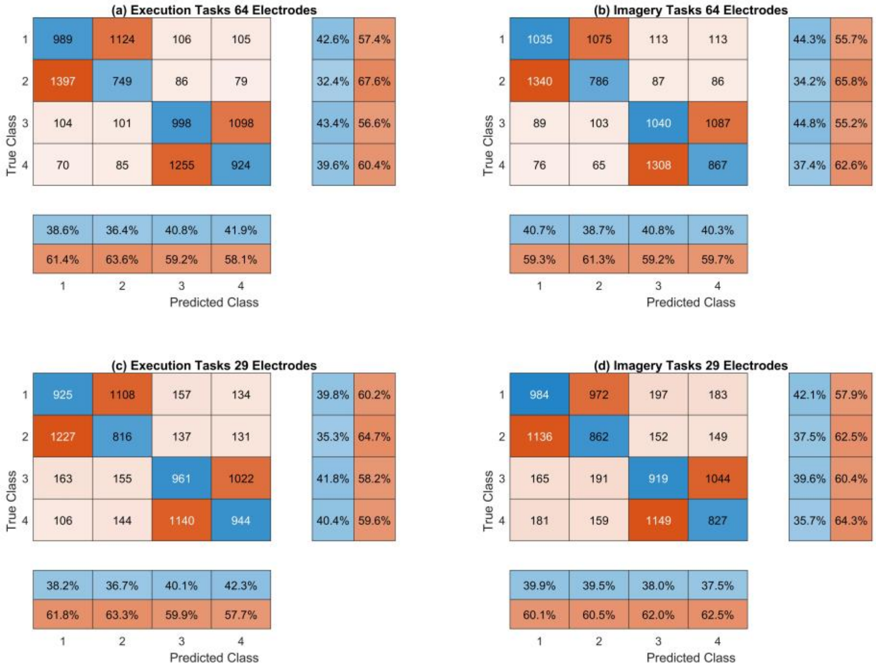

Supplementary Figure S35: FgMDMS Classifier Confusion matrices

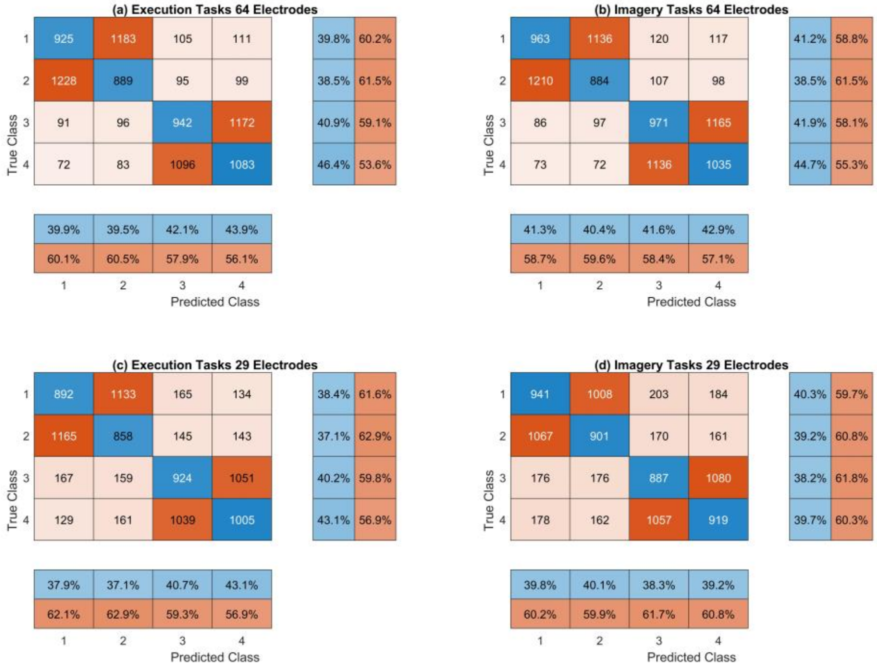

Supplementary Figure S36: FgMDMU Classifier Confusion matrices

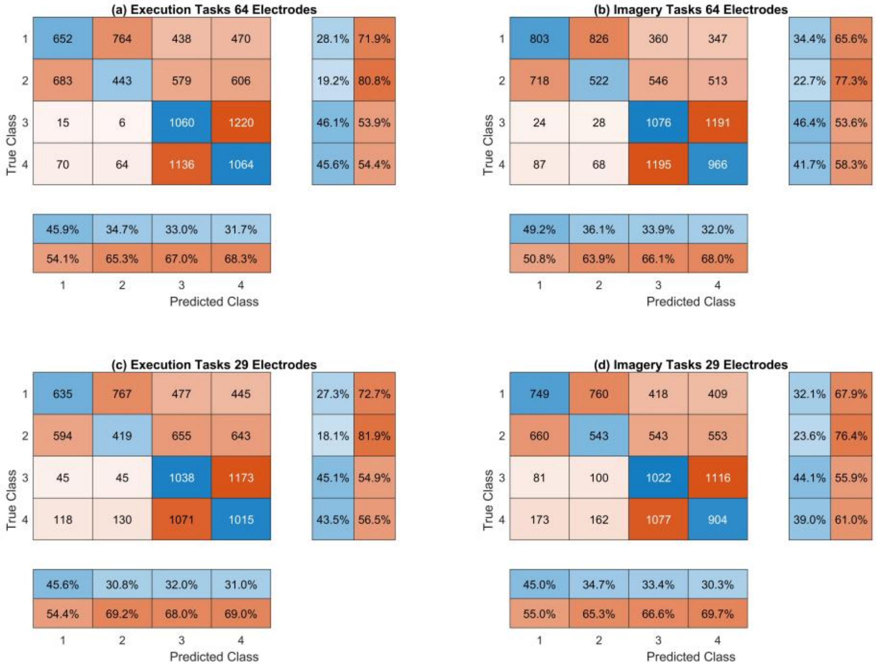

Supplementary Figure S37: FgMDMR Classifier Confusion matrices

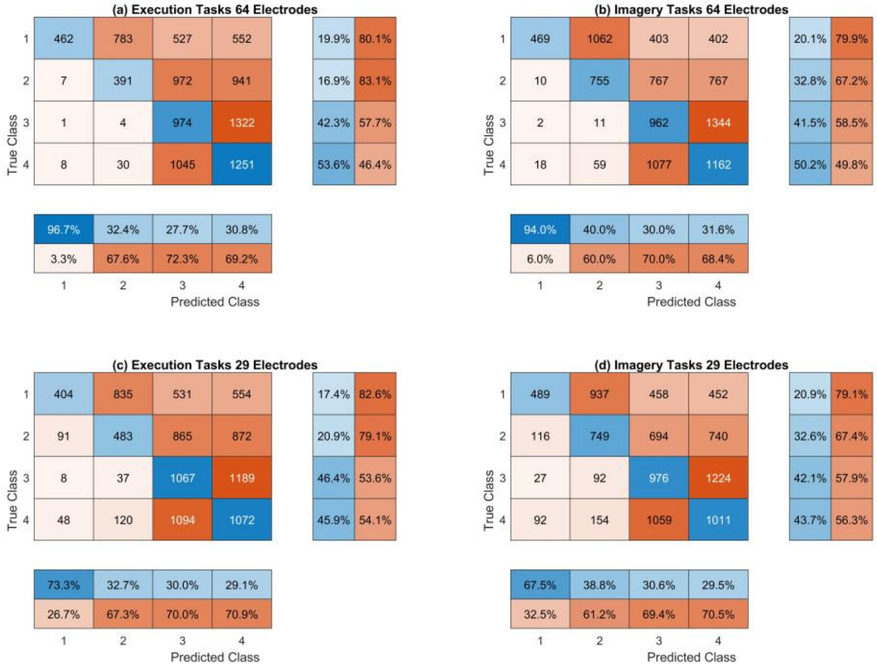

Supplementary Figure S38: FgMDMRS Classifier Confusion matrices

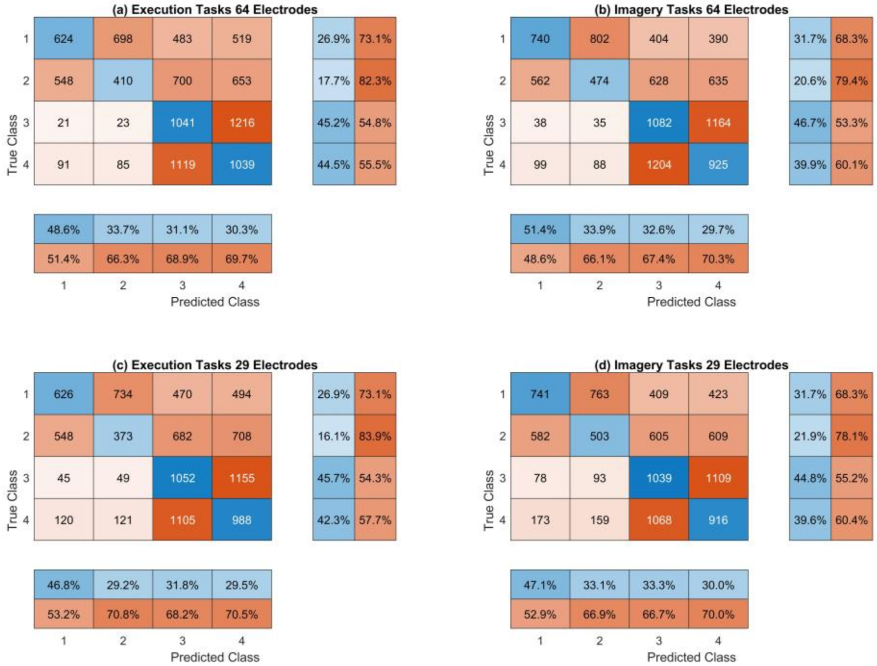

Supplementary Figure S39: FgMDMRU Classifier Confusion matrices

Simple Main Effect and Pairwise Comparison Data Sheets

The following tables summarize the post hoc test (simple main effect). Emphasized values (***bold and italic***) are significant. Red values are negative, i.e. the performance of the classifier in the second column is lower than the performance of the classifier in the top row (tables 1-4) or lower than the performance in the fifth column (tables 5-8). White values men no difference, i.e. 0.000 difference. All values are with a 95% confidence interval and the significance level is $p < .01$. Please note that the classification accuracies are represented as a percent.

Supplementary Table S4: Pairwise comparison of the classifiers when classifying ME Tasks using 29 electrodes.

		Classifier	MDMS	MDMU	MDMR	MDMRS	MDMRU	FgMDM	FgMDMS	FgMDMU	FgMDMR	FgMDMRS	FgMDMRU
	Accuracy	Mean	75.39383	73.96983	31.34845	30.04318	29.63325	39.82737	39.33118	39.68707	33.51671	30.04318	32.78317
Classifier	Mean	Std. Dev.	3.342126	3.660805	4.985203	4.840529	4.329559	6.294564	6.716091	6.074696	5.742214	4.840529	6.073656
MDM	76.48336	3.173769	***1.090 (.536 to 1.643)***	***2.514 (1.797 to 3.230)***	***45.135 (43.193 to 47.076)***	***46.440 (44.568 to 48.313)***	***46.850 (45.076 to 48.625)***	***36.656 (34.493 to 38.819)***	***37.152 (34.837 to 39.468)***	***36.796 (34.659 to 38.933)***	***42.967 (40.966 to 44.967)***	***43.840 (41.642 to 46.038)***	***43.700 (41.364 to 46.037)***
MDMS	75.39383	3.342126		***1.424 (.904 to 1.944)***	***44.045 (42.088 to 46.003)***	***45.351 (43.499 to 47.202)***	***45.761 (43.963 to 47.559)***	***35.566 (33.372 to 37.760)***	***36.063 (33.753 to 38.372)***	***35.707 (33.559 to 37.855)***	***41.877 (39.848 to 43.906)***	***42.751 (40.545 to 44.956)***	***42.611 (40.289 to 44.932)***
MDMU	73.96983	3.660805			***42.621 (40.589 to 44.653)***	***43.927 (42.001 to 45.853)***	***44.337 (42.445 to 46.228)***	***34.142 (31.901 to 36.384)***	***34.639 (32.264 to 37.013)***	***34.283 (32.089 to 36.476)***	***40.453 (38.381 to 42.525)***	***41.327 (39.025 to 43.629)***	***41.187 (38.822 to 43.552)***
MDMR	31.34845	4.985203				***1.305 (.657 to 1.954)***	***1.715 (.940 to 2.490)***	***-8.479 (-11.001 to -5.957)***	***-7.983 (-10.582 to -5.384)***	***-8.339 (-10.737 to -5.941)***	-2.168 (-4.511 to .175)	-1.294 (-4.041 to 1.452)	-1.435 (-4.145 to 1.276)
MDMRS	30.04318	4.840529					.410 (-.390 to 1.210)	***-9.784 (-12.323 to -7.245)***	***-9.288 (-11.875 to -6.701)***	***-9.644 (-12.086 to -7.202)***	***-3.474 (-5.836 to -1.111)***	-2.600 (-5.361 to .162)	***-2.740 (-5.437 to -.043)***
MDMRU	29.63325	4.329559						***-10.194 (-12.674 to -7.714)***	***-9.698 (-12.252 to -7.144)***	***-10.054 (-12.372 to -7.736)***	***-3.883 (-6.171 to -1.596)***	***-3.010 (-5.631 to -.388)***	***-3.150 (-5.775 to -.524)***
FgMDM	39.82737	6.294564							.496 (-.439 to 1.432)	.140 (-.666 to .947)	***6.311 (5.033 to 7.589)***	***7.184 (5.542 to 8.827)***	***7.044 (5.175 to 8.913)***
FgMDMS	39.33118	6.716091								-.356 (-1.365 to .653)	***5.814 (4.467 to 7.161)***	***6.688 (4.949 to 8.428)***	***6.548 (4.55 to 8.546)***
FgMDMU	39.68707	6.074696									***6.170 (4.830 to 7.511)***	***7.044 (5.366 to 8.722)***	***6.904 (4.915 to 8.893)***
FgMDMR	33.51671	5.742214										.874 (-.816 to 2.564)	.734 (-1.114 to 2.581)
FgMDMRS	32.64293	5.558011											-.14 (-1.636 to 1.356)

Supplementary Table S5: Pairwise comparison of the classifiers when classifying MI Tasks using 29 electrodes.

		Classifier	MDMS	MDMU	MDMR	MDMRS	MDMRU	FgMDM	FgMDMS	FgMDMU	FgMDMR	FgMDMRS	FgMDMRU
	Accuracy	Mean	70.49621	69.82743	33.08518	31.86618	31.71514	39.66558	38.74863	39.35273	34.71412	34.7896	34.50919
Classifier	Mean	Std. Dev.	2.69344	2.803703	3.848775	3.409417	3.547084	6.615369	6.76701	6.522667	5.630163	5.583915	5.048402
MDM	72.04957	2.825291	1.553 (.907 to 2.200)	2.222 (1.549 to 2.896)	38.964 (37.446 to 40.483)	40.183 (38.791 to 41.576)	40.334 (38.834 to 41.835)	32.384 (30.212 to 34.556)	33.301 (31.071 to 35.531)	32.697 (30.519 to 34.875)	37.335 (35.421 to 39.250)	37.260 (34.973 to 39.547)	37.540 (35.506 to 39.575)
MDMS	70.49621	2.69344		.669 (.144 to 1.194)	37.411 (35.903 to 38.919)	38.630 (37.261 to 39.999)	38.781 (37.282 to 40.281)	30.831 (28.730 to 32.931)	31.748 (29.618 to 33.877)	31.143 (29.022 to 33.265)	35.782 (33.897 to 37.667)	35.707 (33.517 to 37.896)	35.987 (34.063 to 37.912)
MDMU	69.82743	2.803703			36.742 (35.223 to 38.261)	37.961 (36.555 to 39.368)	38.112 (36.559 to 39.666)	30.162 (28.027 to 32.297)	31.079 (28.900 to 33.258)	30.475 (28.320 to 32.629)	35.113 (33.207 to 37.020)	35.038 (32.805 to 37.271)	35.318 (33.359 to 37.278)
MDMR	33.08518	3.848775				1.219 (.664 to 1.774)	1.370 (.663 to 2.077)	-6.580 (-8.753 to -4.408)	-5.663 (-7.927 to -3.400)	-6.268 (-8.356 to -4.179)	-1.629 (-3.585 to .327)	-1.704 (-4.151 to .742)	-1.424 (-3.623 to .775)
MDMRS	31.86618	3.409417					.151 (-.442 to .744)	-7.799 (-9.979 to -5.620)	-6.882 (-9.147 to -4.618)	-7.487 (-9.585 to -5.388)	-2.848 (-4.738 to -.958)	-2.923 (-5.351 to -.496)	-2.643 (-4.802 to -.484)
MDMRU	31.71514	3.547084						-7.950 (-10.265 to -5.636)	-7.033 (-9.417 to -4.650)	-7.638 (-9.861 to -5.415)	-2.999 (-5.007 to -.991)	-3.074 (-5.615 to -.534)	-2.794 (-5.074 to -.514)
FgMDM	39.66558	6.615369							.917 (-.010 to 1.844)	.313 (-.485 to 1.110)	4.951 (3.630 to 6.273)	4.876 (2.719 to 7.032)	5.156 (3.122 to 7.191)
FgMDMS	38.74863	6.76701								-.604 (-1.769 to .561)	4.035 (2.469 to 5.600)	3.959 (1.947 to 5.971)	4.239 (2.218 to 6.261)
FgMDMU	39.35273	6.522667									4.639 (3.345 to 5.932)	4.563 (2.51 to 6.616)	4.844 (2.818 to 6.869)
FgMDMR	34.71412	5.630163										-.075 (-2.043 to 1.892)	.205 (-1.792 to 2.201)
FgMDMRS	34.7896	5.583915											.28 (-1.175 to 1.736)

Supplementary Table S6: Pairwise comparison of the classifiers when classifying ME Tasks using 64 electrodes.

		Classifier	MDMS	MDMU	MDMR	MDMRS	MDMRU	FgMDM	FgMDMS	FgMDMU	FgMDMR	FgMDMRS	FgMDMRU
	Accuracy	Mean	81.52093	80.70111	80.33435	34.4013	33.29019	32.90183	41.24055	39.48224	41.4132	33.20392	33.59219
Classifier	Mean	Std. Dev.	2.400122	2.872964	3.43636	4.615241	4.339504	4.320785	7.458902	7.814747	6.968785	5.715146	6.443624
MDM	81.52093	2.400122	.820 (.301 to 1.339)	1.187 (.528 to 1.846)	47.120 (45.311 to 48.928)	48.231 (46.552 to 49.909)	48.619 (46.888 to 50.350)	40.280 (37.613 to 42.947)	42.039 (39.251 to 44.826)	40.108 (37.582 to 42.633)	46.796 (44.345 to 49.247)	48.317 (45.955 to 50.679)	47.929 (45.341 to 50.516)
MDMS	80.70111	2.872964		.367 (-.119 to .853)	46.300 (44.372 to 48.228)	47.411 (45.643 to 49.179)	47.799 (45.935 to 49.664)	39.461 (36.726 to 42.195)	41.219 (38.360 to 44.078)	39.288 (36.685 to 41.890)	45.976 (43.488 to 48.465)	47.497 (45.111 to 49.883)	47.109 (44.471 to 49.747)
MDMU	80.33435	3.43636			45.933 (43.919 to 47.947)	47.044 (45.157 to 48.931)	47.433 (45.459 to 49.406)	39.094 (36.267 to 41.921)	40.852 (37.909 to 43.796)	38.921 (36.233 to 41.609)	45.609 (43.030 to 48.189)	47.130 (44.563 to 49.698)	46.742 (44.019 to 49.465)
MDMR	34.4013	4.615241				1.111 (.450 to 1.772)	1.499 (.584 to 2.415)	-6.839 (-9.674 to -4.004)	-5.081 (-8.089 to -2.072)	-7.012 (-9.671 to -4.353)	-.324 (-2.885 to 2.238)	1.197 (-1.572 to 3.967)	.809 (-2.077 to 3.695)
MDMRS	33.29019	4.339504					.388 (-.422 to 1.199)	-7.950 (-10.752 to -5.149)	-6.192 (-9.180 to -3.204)	-8.123 (-10.755 to -5.491)	-1.435 (-3.977 to 1.108)	.086 (-2.611 to 2.783)	-.302 (-3.195 to 2.591)
MDMRU	32.90183	4.320785						-8.339 (-11.168 to -5.509)	-6.580 (-9.554 to -3.607)	-8.511 (-11.192 to -5.831)	-1.823 (-4.353 to .707)	-.302 (-3.038 to 2.434)	-.69 (-3.536 to 2.155)
FgMDM	41.24055	7.458902							1.758 (.729 to 2.787)	.173 (-1.082 to .737)	6.516 (5.131 to 7.900)	8.037 (5.891 to 10.182)	7.648 (5.362 to 9.934)
FgMDMS	39.48224	7.814747								-1.931 (-3.040 to -.822)	4.757 (3.408 to 6.107)	6.278 (4.149 to 8.408)	5.890 (3.678 to 8.102)
FgMDMU	41.4132	6.968785									6.688 (5.284 to 8.093)	8.209 (6.061 to 10.357)	7.821 (5.507 to 10.135)
FgMDMR	34.72492	6.702413										1.521 (-.407 to 3.449)	1.133 (-.833 to 3.098)
FgMDMRS	33.20392	5.715146											-.388 (-1.916 to 1.139)

Supplementary Table S7: Pairwise comparison of the classifiers when classifying MI Tasks using 64 electrodes.

		Classifier	MDMS	MDMU	MDMR	MDMRS	MDMRU	FgMDM	FgMDMS	FgMDMU	FgMDMR	FgMDMRS	FgMDMRU
	Accuracy	Mean	75.07022	74.76815	35.74971	34.80039	34.51988	41.44552	40.21578	41.56418	36.32148	36.11656	34.746553
Classifier	Mean	Std. Dev.	2.971214	3.22872	3.532345	3.437841	3.057918	7.614004	7.623842	7.024581	6.413133	6.269702	6.106047
MDM	76.41869	3.354806	1.348 (.817 to 1.880)	1.651 (1.058 to 2.243)	40.669 (38.961 to 42.377)	41.618 (39.940 to 43.297)	41.899 (40.247 to 43.551)	34.973 (32.116 to 37.831)	36.203 (33.310 to 39.096)	34.855 (32.142 to 37.567)	40.097 (37.618 to 42.576)	40.302 (37.595 to 43.009)	41.672 (39.043 to 44.302)
MDMS	75.07022	2.971214		.302 (-.141 to .745)	39.321 (37.698 to 40.943)	40.270 (38.673 to 41.866)	40.550 (38.977 to 42.124)	33.625 (30.860 to 36.389)	34.854 (32.068 to 37.641)	33.506 (30.883 to 36.129)	38.749 (36.371 to 41.126)	38.954 (36.314 to 41.593)	40.324 (37.738 to 42.91)
MDMU	74.76815	3.22872			39.018 (37.290 to 40.747)	39.968 (38.256 to 41.680)	40.248 (38.587 to 41.910)	33.323 (30.490 to 36.155)	34.552 (31.686 to 37.419)	33.204 (30.507 to 35.901)	38.447 (35.971 to 40.923)	38.652 (35.952 to 41.351)	40.022 (37.397 to 42.646)
MDMR	35.74971	3.532345				.949 (.356 to 1.543)	1.230 (.457 to 2.003)	-5.696 (-8.310 to -3.081)	-4.466 (-7.076 to -1.856)	-5.814 (-8.258 to -3.371)	-.572 (-2.897 to 1.754)	-.367 (-3.11 to 2.376)	1.003 (-1.541 to 3.547)
MDMRS	34.80039	3.437841					.281 (-.528 to 1.089)	-6.645 (-9.333 to -3.957)	-5.415 (-8.078 to -2.753)	-6.764 (-9.270 to -4.257)	-1.521 (-3.902 to .860)	-1.316 (-4.127 to 1.495)	.054 (-2.558 to 2.666)
MDMRU	34.51988	3.057918						-6.926 (-9.590 to -4.261)	-5.696 (-8.367 to -3.025)	-7.044 (-9.534 to -4.555)	-1.802 (-4.091 to .488)	-1.597 (-4.319 to 1.125)	-.227 (-2.685 to 2.232)
FgMDM	41.44552	7.614004							1.230 (.362 to 2.098)	-.119 (-.901 to .664)	5.124 (3.753 to 6.495)	5.329 (2.518 to 8.139)	6.699 (4.11 to 9.288)
FgMDMS	40.21578	7.623842								-1.348 (-2.484 to -.213)	3.894 (2.491 to 5.298)	4.099 (1.295 to 6.903)	5.469 (2.882 to 8.057)
FgMDMU	41.56418	7.024581									5.243 (3.895 to 6.590)	5.448 (2.755 to 8.14)	6.818 (4.326 to 9.309)
FgMDMR	36.32148	6.413133										.205 (-2.194 to 2.604)	1.575 (-.612 to 3.762)
FgMDMRS	36.11656	6.269702											1.37 (-.283 to 3.023)

Supplementary Table S8: Pairwise comparison of different classifier adaptation strategies: ME vs. MI when using 29 electrodes.

	ME 29 Elect.		Pairwise Comparison	MI 29 Elect.	
Classifier	Mean	Std.		Mean	Std.
MDM	76.483	3.174	**4.434 (3.603 to 5.265)**	72.050	2.825
MDMS	75.394	3.342	**4.898 (4.128 to 5.667)**	70.496	2.693
MDMU	73.970	3.661	**4.142 (3.287 to 4.998)**	69.827	2.804
MDMR	31.348	4.985	**-1.737 (-2.927 to -0.547)**	33.085	3.849
MDMRS	30.043	4.841	**-1.823 (-2.924 to -0.722)**	31.866	3.409
MDMRU	29.633	4.330	**-2.082 (-3.071 to -1.093)**	31.715	3.547
FgMDM	39.827	6.295	0.162 (-1.586 to 1.910)	39.666	6.615
FgMDMS	39.331	6.716	0.583 (-1.128 to 2.293)	38.749	6.767
FgMDMU	39.687	6.075	0.334 (-1.336 to 2.005)	39.353	6.523
FgMDMR	33.517	5.742	-1.197 (-2.673 to 0.278)	34.714	5.630
FgMDMRS	32.643	5.558	**-2.147 (-3.897 to -0.397)**	34.790	5.584
FgMDMRU	32.783	6.074	-1.726 (-3.638 to 0.186)	34.509	5.048

Supplementary Table S10: Pairwise comparison of different classifier adaptation strategies: 29 electrodes vs. 64 electrodes for ME trials.

	ME 29 Elect.		Pairwise Comparison	ME 64 Elect.	
Classifier	Mean	Std.		Mean	Std.
MDM	76.483	3.174	**-5.038 (-5.717 to -4.358)**	81.521	2.400
MDMS	75.394	3.342	**-5.307 (-6.048 to -4.567)**	80.701	2.873
MDMU	73.970	3.661	**-6.365 (-7.269 to -5.460)**	80.334	3.436
MDMR	31.348	4.985	**-3.053 (-4.224 to -1.882)**	34.401	4.615
MDMRS	30.043	4.841	**-3.247 (-4.391 to -2.103)**	33.290	4.340
MDMRU	29.633	4.330	**-3.269 (-4.324 to -2.214)**	32.902	4.321
FgMDM	39.827	6.295	-1.413 (-2.831 to 0.005)	41.241	7.459
FgMDMS	39.331	6.716	0.151 (-1.515 to 1.213)	39.482	7.815
FgMDMU	39.687	6.075	**-1.726 (-3.094 to -.358)**	41.413	6.969
FgMDMR	33.517	5.742	-1.208 (-2.550 to .134)	34.725	6.702
FgMDMRS	32.643	5.558	-0.561 (-2.157 to 1.035)	33.204	5.715
FgMDMRU	32.783	6.074	-0.809 (-2.528 to 0.910)	33.592	6.444

Supplementary Table S9: Pairwise comparison of different classifier adaptation strategies: ME vs. MI when using 64 electrodes.

	ME 64 Elect.		Pairwise Comparison	MI 64 Elect.	
Classifier	Mean	Std.		Mean	Std.
MDM	81.521	2.400	**5.102 (4.501 to 5.704)**	76.419	3.355
MDMS	80.701	2.873	**5.631 (5.020 to 6.242)**	75.070	2.971
MDMU	80.334	3.436	**5.566 (4.946 to 6.187)**	74.768	3.229
MDMR	34.401	4.615	**-1.348 (-2.442 to -0.255)**	35.750	3.532
MDMRS	33.290	4.340	**-1.510 (-2.576 to -0.445)**	34.800	3.438
MDMRU	32.902	4.321	**-1.618 (-2.656 to -0.580)**	34.520	3.058
FgMDM	41.241	7.459	-0.205 (-1.838 to 1.429)	41.446	7.614
FgMDMS	39.482	7.815	-0.734 (-2.470 to 1.003)	40.216	7.624
FgMDMU	41.413	6.969	-0.151 (-1.644 to 1.342)	41.564	7.025
FgMDMR	34.725	6.702	**-1.597 (-3.071 to -.122)**	36.321	6.413
FgMDMRS	33.204	5.715	**-2.913 (-5.131 to -0.694)**	36.117	6.270
FgMDMRU	33.592	6.444	-1.154 (-3.324 to 1.0152)	34.747	6.106

Supplementary Table S11: Pairwise comparison for different classifier adaptation strategies: 29 electrodes vs. 64 electrodes for MI trials.

	MI 29 Elect.		Pairwise Comparison	MI 64 Elect.	
Classifier	Mean	Std.		Mean	Std.
MDM	72.050	2.825	**-4.369 (-5.139 to -3.600)**	76.419	3.355
MDMS	70.496	2.693	**-4.574 (-5.346 to -3.802)**	75.070	2.971
MDMU	69.827	2.804	**-4.941 (-5.760 to -4.121)**	74.768	3.229
MDMR	33.085	3.849	**-2.665 (-3.709 to -1.620)**	35.750	3.532
MDMRS	31.866	3.409	**-2.934 (-3.871 to -1.997)**	34.800	3.438
MDMRU	31.715	3.547	**-2.805 (-3.730 to -1.879)**	34.520	3.058
FgMDM	39.666	6.615	**-1.780 (-3.281 to -.279)**	41.446	7.614
FgMDMS	38.749	6.767	-1.467 (-3.007 to .073)	40.216	7.624
FgMDMU	39.353	6.523	**-2.211 (-3.625 to -.798)**	41.564	7.025
FgMDMR	34.714	5.630	**-1.607 (-2.879 to -.336)**	36.321	6.413
FgMDMRS	34.790	5.584	-1.327 (-3.114 to 0.461)	36.117	6.270
FgMDMRU	34.509	5.048	-0.237 (-1.967 to 1.492)	34.747	6.106
